# Ribonucleases in Mendelian disease: Characterization and insight from model organisms

**DOI:** 10.1016/j.gendis.2025.101613

**Published:** 2025-03-25

**Authors:** Annasha Dutta, Anastasiia Zaremba, Paulina Jackowiak

**Affiliations:** Institute of Bioorganic Chemistry Polish Academy of Sciences, Noskowskiego 12/14 61-704 Poznań, Poland

**Keywords:** Evolution, Mendelian disorders, Metabolism, Model organisms, Mutations, Ribonucleases

## Abstract

Ribonucleases (RNases), essential for RNA metabolism, are implicated in human diseases, including neurodevelopmental, developmental, hematopoietic and other dysfunctions through mutations that disrupt their enzymatic functions. Exploring RNase mutations across organisms offers insights into Mendelian diseases, facilitating molecular dissection of pathological pathways and therapeutic development. By employing model organisms, our analysis underscores the evolutionary conservation of RNase genes, facilitating deeper insights into disease mechanisms. These models are vital for uncovering rare molecular dysfunctions and potential therapeutic targets, demonstrating the effectiveness of integrated research approaches in addressing complex genetic disorders. Drawing from phylogenetic analyses, literature survey, and databases documenting the effects of human disease-causing mutations, the review highlights the significance and advantages of employing model organisms to study specific Mendelian disorders.

## Introduction

Ribonucleases (RNases) constitute a diverse group of enzymes that play a predominant role in RNA metabolism, encompassing degradation, steady-state turnover, quality control, and maturation processes. RNases can be classified into exoribonucleases and endoribonucleases based on their mode of action. Exoribonucleases cleave RNA molecules from either the 3′ or 5′ terminal, predominantly releasing nucleoside monophosphates, though in rare cases, nucleoside diphosphates may also be produced. There are six major superfamilies of exoribonucleases, including RNR, DEDD, RBN, PDX, RRP4, and 5PX, with DEDD being the largest and most diverse group.^1^ Endoribonucleases, on the other hand, cleave RNA substrates internally, releasing RNA fragments of various lengths. Eukaryotic endoribonucleases comprise a large group of enzymes from diverse families, such as RNase A, RNase L, RNase III, MBL, RNase H, Schlafen, and multi-protein complexes like RNase P/MRP.^2^ The classification of RNases based on the mechanism of action and released products, on the other hand, has been a subject of contention. The current description by the Biochemical Nomenclature Committee of IUPAC and NC-IUBMB categorizes them as either hydrolases acting on ester bonds (EC 3.1) that cleave phosphodiester bonds to produce 5′ or 3′ monoesters, or phosphorous oxygen lyases (EC 4.6.1), which cleave the same phosphodiester bonds to produce 2′,3′-cyclophosphate intermediates and 3′-phosphate as end-products.^3^

While each RNase plays a unique and important role in global cell homeostasis, specifically some are of clinical interest due to their established links to sporadic diseases like cancers^4^ and a range of hereditary Mendelian disorders listed in the Online Mendelian Inheritance in Man (OMIM) database.^5^ Mutations, whether directly within the catalytic domain or in any other key motif, are often ultimately linked to a compromised catalytic activity of the protein. Moreover, the genetic alterations have the potential to affect the transcript encoding an RNase or the enzyme itself, a topic which has been briefly discussed with suitable examples, later in the review. Given their critical roles in maintaining cellular homeostasis, it is evident that the disease-associated RNases, along with many others, are widely distributed across various kingdoms of life, with their key motifs being highly conserved. When discussing Mendelian disorders, their rarity in the population restricts exploratory studies on causal mutations, leading to many alterations going unnoticed. However, keeping in mind the ever-increasing amounts of genetic and genomic data, it has been suggested that an additional 6000 to 13,000 genes shall be added to the list of genes associated with monogenic hereditary disorders in the future.^6^ A common approach to studying and characterizing previously undiagnosed or rarely diagnosed diseases involves analyzing known or novel high-risk genetic variants and their associated molecular phenotypes at the level of non-mammalian model organisms. The conserved nature of RNases indeed broadens the scope for modeling RNase-associated Mendelian diseases in non-mammalian systems, enabling deeper exploration of their molecular mechanisms. Model organisms eliminate the dependency of such studies on the availability of rare patient samples. The significance of models in rare disease research has surged globally, particularly with the establishment of the NIH-common-funded Undiagnosed Diseases Network(UDN). This initiative includes a Model Organism Screening Center (MOSC) dedicated to studies on *Caenorhabditis elegans* (nematode), *Drosophila melanogaster* (fly), and *Danio rerio* (fish), among other resources.^7^

Building upon the existing knowledge base, this review delves into the molecular pathologies of RNase-associated hereditary Mendelian diseases. In doing so, we present updated information on disease models, drawing from both an in-house conducted phylogenetic study and available literature. Additionally, we highlight the significance and advantages of modeling rare genetic disorders in non-mammals. This approach offers valuable insights into the underlying molecular perturbations which shall aid in the development of future therapeutic strategies.

## Different types of mutations disrupting RNase functions

The physiological function of an RNase can be altered via various indirect and direct pathways. Alterations can occur within or outside the protein-coding region. Such mutations thus can directly affect the catalytic activity or indirectly affect a regulatory pathway which in turn fine-tunes the expression and action of the RNase. Below, we illustrate various types of mutations by drawing examples from the RNase group of proteins found across multiple kingdoms of life.

The protein-coding part of a gene is the region that is both transcribed and translated to finally synthesize the polypeptide sequence which undergoes further post-translational modifications and folding events to form the functional protein. In most cases, mutations in such regions directly alter the amino acid sequence affecting the protein function through multiple means, as depicted by the following examples.

Mutations in one or all three subunits of the RNase H2 enzyme (H2A, H2B, and H2C) cause the genetic disorder called Aicardi-Goutières syndrome (AGS). A missense mutation in the catalytic subunit RNase H2A coding gene causes severely reduced enzymatic activity leading to a specific subtype of the disease.^8^ In the yeast RNase III enzyme Rnt1p, depending on the type of mutation within the dsRNA binding domain, the function of the enzyme is impeded to varying degrees*.* The effects range from disruption of enzyme–substrate interaction to differential dsRNA processing defects, *in vivo* and/or *in vitro*.^9^ Mutations that impact protein folding can be exemplified by *Escherichia coli* RNase HI. Substitution mutations in the enzymatic core perturb the physiological balance between folding intermediates and native folded proteins. Such mutations can either lead to the accumulation or destabilization of folding intermediates, which further interfere with the proper folding of the mature enzyme.^10^ Mutations can sometimes change the subcellular localization of an enzyme. For example, a single mutation in the N-terminal domain of the RNase III enzyme in yeast, Rnt1p, is sufficient to prevent its accumulation in the nucleoplasm leading to slower exit from mitosis.^11^ Certain highly pathogenic mutations in RNase coding genes can significantly alter their RNA transcripts. For instance, a homozygous stop-gain mutation in the PARN-like ribonuclease domain containing exonuclease 1 (PNLDC1) gene leads to the production of abnormal transcripts, which are subsequently degraded through the nonsense-mediated RNA decay (NMD) pathway. This process ultimately results in a complete loss of protein function.^12^

## Human RNases implicated in Mendelian diseases

Mendelian or monogenic diseases are caused by the mutation of a single gene and run through generations. The disease-associated gene could be located on an autosome or the sex chromosome and can be dominant or recessive in terms of phenotypic manifestations. The diseases are categorized as follows: autosomal dominant/recessive; X-linked dominant/recessive, and Y-linked.^13^

Currently, UniProt enlists 104 RNases, of which 13 proteins are implicated in hereditary Mendelian disorders. But with the advances made in the field of creation of viable organisms carrying lethal gene knockouts using CRISPR-Cas9 method in a 2-cell embryonic system,^14^ it is likely that essential RNases crucial for viability will be progressively identified and studied. This could lead to the discovery of various hereditarily transferred lethal mutations in essential RNases, providing new subjects for research.

**Figure 1:**
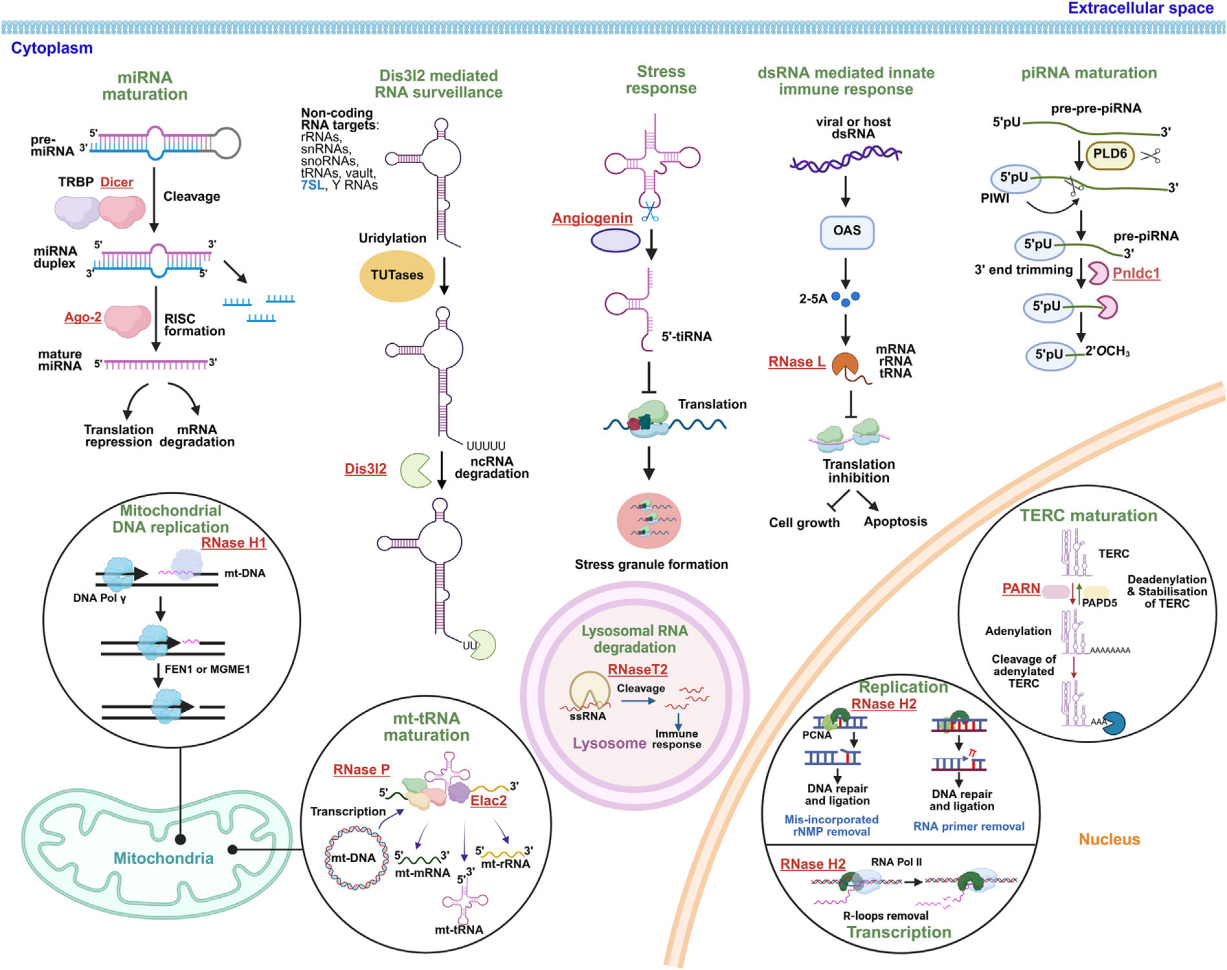
Non-coding RNA metabolism pathways disrupted upon mutations of the 12 selected RNases implicated in Mendelian diseases. SLFN14 was excluded as its mechanism of action has not yet been fully elucidated.

When examining diseases caused by disorders in the genes encoding RNases, an interesting pattern can be noticed, that is, most of them are neurological or growth-related diseases. This therefore raises the question about the existence of certain similarities in disease pathogenesis resulting from the common properties of these RNases. To provide an insight into this phenomenon, we therefore present below the key features of individual RNases in the context of the main groups of diseases associated with them. The cellular processes mediated by these RNases are shown in Figure 1. The characteristics and molecular details of the genes encoding these enzymes are summarized in Table 1.

**Table 1:** Human ribonuclease genes associated with Mendelian disorders.

Gene name	Protein name	RNase family	Substrate	Cellular localization	OMIM ID	Disease name	Disease type	Disease onset	Disease mechanism- current view
*RNASEH2A*	Ribonuclease H2 subunit A (RNase H2A)	RNase H family/Retrovirus Integrase Superfamily (RISF)	RNA:DNA hybrids	Nucleus	610333	Aicardi-Goutières syndrome 4	Neurological	Early	Mutations causing loss of enzyme function lead to chronic DNA damage and replication stress. This triggers severe immunogenic response in the neuronal tissue causing Aicardi-Goutières syndrome (AGS). Chronic DNA damage affects the central nervous system because the bulk of the tissue is comprised of long-living neurons for which genome maintenance is of utmost importance.
*RNASEH2B*	Ribonuclease H2 subunit B (RNase H2B)			Nucleus	610181	Aicardi-Goutières syndrome 2	Neurological	Early	
*RNASEH2C*	Ribonuclease H2 subunit C (RNase H2C)			Nucleus	610329	Aicardi-Goutières syndrome 3	Neurological	Early	
*RNASE5*	Angiogenin	RNase A family	Mature tRNA; snRNAs at specific sites; miRNA	Nucleus; cytoplasm	611895	Amyotrophic lateralsclerosis 9	Neurological	Adulthood	Angiogenin curbs stress by generating stress-induced tRNA fragments called 5′- tiRNA that reprogram global protein translation thereby promoting cell survival. In ALS, mutation of the gene causes progressive motor neuron death, where it is also highly expressed. The effect is specific to motor neurons because they are morphologically very long and highly active cells, for which localized stress protection by angiogenin via decentralized translation control is crucial.
*AGO2*	Protein argonaute-2 (Ago-2)	RNase H family/Retrovirus Integrase Superfamily (RISF)	miRNA/siRNA: Target RNA hybrids	Nucleus; cytoplasm	619149	Lessel-Kreienkampsyndrome	Neurological	Early	Disease-causing AGO2 mutations result from the prolonged binding of the protein onto target mRNAs in the RNA silencing pathway. This leads to sequestration of Ago-2 protein in dendritic P-bodies further hampering RNA-silencing mediated translational regulation in neurons. Its function is especially important in neurons as much of their dynamic functions rely on compartmentalized translation.
*RNASET2*	Ribonuclease T2 (RNase T2)	RNase T2 family	rRNA, mRNA	Lysosomes; mitochondria; vacuoles; P-bodies	612951	Cystic leukoencephalopathy, without megalencephaly	Neurological	Early	The lysosomal enzyme is active against ssRNA, especially rRNA. As the gene is highly expressed in the brain, deficiency of the enzyme causes an overt immune response in the brain similar to that of congenital cytomegalovirus infection or AGS in the central nervous system. This immune reaction is due to the accumulation of undigested ssRNAs in the lysosome and their transport to cytosol where they bind to RNA-recognizing immune receptors.
*DICER1*	Endoribonuclease Dicer (Dicer)	RNase III family	Premature miRNAs (pri-miRNA)	Nucleus; cytoplasm	618272; 138800; 601200; 180295	GLOW syndrome, somatic mosaic; multinodular goiter 1, with or without Sertoli-Leydig cell tumors; pleuropulmonary blastoma; embryonal rhabdomyosarcoma 2	Growth-related	Early	The enzyme is involved in the generation of 3p and 5p-miRNAs. Molecularly, DICER1 mutations in a specific region result in the diminishing of 5p-miRNAs and accumulation of 3p-miRNAs. Most of the aberrantly accumulated 3p-miRNA molecules negatively regulate inhibitors and positively regulate activators of the central PI3K-AKT-mTOR growth signaling pathway. This results in the manifestation of overgrowth or abnormal growth of multiple organs.
*DIS3L2*	DIS3-like exonuclease 2 (Dis3l2)	RNase II family (component of RNA exosome complex)	rRNAs, snRNAs, snoRNAs, tRNAs, vault, 7SL, Y RNAs, mRNAs, lncRNAs, transcripts from pseudogenes	Cytoplasm	267000	Perlman syndrome	Growth-related	Early	Dis3l2-mediated decay is a surveillance pathway for a number of coding and non-coding RNAs. Disease-causing mutations in *DIS3L2* cause the accumulation of aberrant 3′ uridylated 7SL ncRNA component of signal recognition particle (SRP). Disturbed quality control of 7SL ncRNA harms ER-associated translation and disturbs cellular calcium homeostasis by endoplasmic reticulum calcium leakage.
*ELAC2*	Zinc phosphodiesterase ELAC protein 2 (Elac2)	RNase Z family/MBL superfamily	tRNA precursors (pre-tRNA); miRNA; piRNA; snRNA; snoRNA	Nucleus; mitochondria	615440; 614731	Combined oxidative phosphorylation deficiency 17; susceptibility to hereditary prostate cancer 2	Growth-related; prostate cancer	Early	Elac2 is involved in the 3′ end processing of pre-tRNAs. Deficiency of the functional enzyme leads to the accumulation of unprocessed mt-tRNA transcripts and disruption of mitochondrial protein translation, especially of the mitochondrially encoded OXPHOS proteins. This leads to a mitochondrial disorder with a deficiency in ATP production, affecting multiple organs.
*PRORP*	Mitochondrial ribonuclease P catalytic subunit (MRPP3)	Components of RNase P riboprotein complex	tRNA precursors (pre-tRNA); mRNA with modified 3′ external guide sequence	Nucleus; mitochondria	619737	Combined oxidative phosphorylation deficiency 54	Growth-related	Early	This protein is the catalytic subunit of mitochondrial RNase P which processes 5′ ends of pre-tRNA molecules in the maturation pathway of functional tRNA. Loss of function of this subunit leads to the deficit of mature mt-tRNAs inhibiting translation of mitochondrial proteins, like several OXPHOS pathway components. This finally causes mitochondrial dysfunction affecting multiple organs.
*PARN*	Poly(A)-specific ribonuclease PARN (PARN)	CAF family	rRNA; snoRNA; scaRNA; mRNA; TERC	Shuffles between nucleus and cytoplasm	616353; 616371	Autosomal recessive dyskeratosis congenita 6; telomere-related pulmonary fibrosis and/or bone marrow failure syndrome 4	Blood-related	Early	The enzyme is involved in the biogenesis of TERC (telomerase RNA component). It stabilizes TERC by removing 3′ poly-A tails that serve as RNA degradation signals and mutation of the gene destabilizes this process. Maintenance of telomere lengths by telomerase is crucial for the survival of various stem and progenitor cell populations like hematopoietic stem cells. Hence, the bone marrow is susceptible to telomeropathies.
*SLFN14*	Protein SLFN14 (SLFN14)	Schlafen family	rRNA	Nucleus	616913	Platelet-type bleeding disorder 20	Blood-related	Early	Platelets and erythrocytes have minimal RNA load. The protein is highly expressed in immature platelets and erythrocytes called megakaryocytes and reticulocytes, respectively. It associates with ribosomes and degrades rRNA to clear RNA during blood cell maturation. Therefore, loss of SLFN14 protein function could hamper the blood cell maturation process causing bleeding disorder.
*RNASEH1*	Ribonuclease H1 (RNase H1)	RNase H family/retrovirus integrase superfamily (RISF)	RNA:DNA hybrids	Nucleus; mitochondria	616479	Autosomal recessive progressive external ophthalmoplegia with mitochondrial DNA deletions 2	Muscular	Adulthood	The enzyme removes RNA primers forming RNA:DNA hybrids in mtDNA replication. Loss of RNase H1 function stalls mtDNA replication fork causing mtDNA depletions and deletions and resultant mitochondrial dysfunction. The major tissue system affected are muscles as they are known to have high energy requirements.
*RNASEL*	2-5A-dependent ribonuclease (RNase L)	Ribonuclease 2–5 A or RNase L family	Microbial rRNA, mRNA	Nucleus; cytoplasm; mitochondria	601518	Prostate cancer 1	Prostate cancer	Adulthood	RNase L is activated by cleavage product of an interferon-induced enzyme called 2′–5′ oligoadenylate synthetase (OAS). Activated RNase L cleaves 28S and 18S rRNA causing cellular apoptosis. This pro-apoptotic activity is both anti-viral and anti-tumorigenic (apoptosis of prostate cancer cells). Prostate cancer is especially implicated in RNase L mutations because the gene maps to hereditary-prostate-cancer (HPC)–predisposition locus (*HPC1*).
*PNLDC1*	Poly(A)-specific ribonuclease (Pnldc1)	CAF family	mRNA; pre-piRNA	Cytoplasm	619528	Spermatogenic failure 57	Reproductive failure	Adulthood	The protein trims 3′ ends of pre-piRNAs, required as a step for piRNA biogenesis. Mature piRNAs are essential in the context of transposon silencing and regulation of spermatogenesis. Therefore, dysfunction of the enzyme leads to the accumulation of 3′-untrimmed piRNAs and de-repression of transposons in the testes causing failure of sperm production.

## Neurological disorders

The nervous system poses both as a sensitive and robust tissue system in the face of genetic insults occurring throughout an organism's lifespan. Hereditary mutation in a single RNase encoding gene is enough to cause an array of neurological malfunctions which are often elicited as neurodevelopmental, neurodegenerative, and/or neuroinflammatory defects. The group of RNases whose mutations lead to various neurological diseases include ribonuclease H2 subunits A, B, and C (RNase H2), angiogenin, argonaute-2 (Ago-2), and ribonuclease T2 (protein referred to here as RNase T2).

RNase H2 holoenzyme is composed of three subunits: RNase H2A being the catalytic subunit, and RNase H2B and H2C, the non-catalytic structural subunits.^15^ In humans, the genes encoding each of the subunits are located on different chromosomes, *RNASEH2A* on 19, *RNASEH2B* on 13, and *RNASEH2C* on 11. The enzyme acts as a surveillance agent removing mis-incorporated ribonucleotides from DNA and resolving DNA:RNA hybrids, formed during replication and transcription, mainly in the nucleus.^16^^,^^17^ Heterozygous loss of function (LOF) mutations in either of the three subunits are associated with a severe autosomal recessive neuroinflammatory disorder AGS, as briefly stated before (AGS2 (RNase H2A); AGS3 (RNase H2B) and AGS4 (RNase H2C)). Mutations in the genes coding for these three subunits have been mapped within their respective protein coding regions.^18^ The disease is manifested by encephalopathy with calcification of the basal ganglia and abnormalities of cerebrospinal fluid that include lymphocytosis and high levels of interferon-α.^19^^,^^20^ Depending on which subunit of the holoenzyme is affected by a mutation, the phenotype of the disorder has a neonatal (*RNASEH2A* and *RNASEH2C*) or later (*RNASEH2B*) onset. The molecular mechanism of AGS involves chronic DNA damage caused by the accumulation of mis-incorporated ribonucleotides, leading to an excessive immune response.^21^ However, the exact DNA damage pathway triggered by these progressively accumulating ribonucleotides remains to be clearly elucidated. Moreover, the link between DNA damage and inflammation phenotype has not been fully resolved yet.

Angiogenin is a secreted enzyme with relatively weak catalytic activity and its *in vivo* substrates have been only partially characterized.^22^ It is encoded by the *RNASE5* gene, which is located on chromosome 14 in humans. In physiological conditions, the enzyme is associated with angiogenesis via the promotion of cell growth, proliferation, and survival. During growth, angiogenin is translocated into the nucleus and acts as a transcription factor that binds rDNA promoters, thus stimulating rRNA synthesis. This is a rate-limiting step in ribosome biogenesis, which in turn is required for translation and other growth-promoting functions.^23^ During stress, on the other hand, angiogenin localizes to the cytoplasm to generate stress-induced tRNA fragments called 5′-tiRNA. These molecules trigger the formation of stress granules and inhibit the global translation of housekeeping genes. Elsewhere, angiogenin might promote the translation of certain anti-apoptotic mRNAs like Bcl-2 during adverse conditions. All angiogenin's stress-responsive functions, taken together, ultimately promote cell survival.^24^^,^^25^ Heterozygous LOF mutations in the *RNASE5* gene are associated with a familial form of amyotrophic lateral sclerosis (ALS), characterized by an onset 10–15 years earlier than ALS cases linked to mutations in other genes.^26^ All ALS-causing mutations in *RNASE5* identified so far have been mapped within the protein-coding region of the gene and cause decreased enzymatic activity.^27^ Major mutations, such as substitutions of key residues His13 and/or His114, lead to a 10,000-fold decrease in activity.^22^ The major phenotype of this disease is a progressive loss of motor neurons in the brain, brainstem, and spinal cord.^28^ It is associated with insufficient rRNA synthesis^23^ and the disturbance of tiRNA generation, followed by impairment of neuroprotective pathways.^29^ However, the link between the molecular phenotype and the brain-centric disease pathology remains to be determined.

Ago-2 encoded by the *AGO2* gene located on chromosome 8, is the only member of the argonaute protein family with important catalytic activities. The protein has the characteristic PIWI and PAZ domains of the family. Its major function is the cleavage of target mRNAs guided by miRNA and siRNA, as a part of the RISC complex.^30^ Within the RNA silencing pathway, it can also process premature miRNAs in a Dicer independent way.^31^ LOF heterozygous mutations in *AGO2* cause the autosomal dominant Lessel-Kreienkamp syndrome (LESKRES). This disorder is manifested predominantly with developmental delay, as well as intellectual and motor disability. The disease-causing mutations are located in the protein-coding region and all the resultant protein variants are inefficient effectors of the shRNA-mediated RNA silencing. One of the key abnormalities associated with LESKRES-causing mutations is the unavailability of free forms of Ago-2, causing global impairment of RNA interference and profound transcriptomic changes. This mainly occurs as a result of the mutated protein getting stalled at mRNA:miRNA hybrids which in turn are then increasingly stacked into RNA degradative granules called P-bodies.^32^ The precise identification of key events leading to the development of the pleiotropic disease phenotype however requires further research.

All the above three RNases are crucial for survival, and homozygous null mutations or knockouts of their coding genes are lethal in nature.^32^, ^33^, ^34^

Ribonuclease T2 from the RNase T2 family is a lysosomal enzyme encoded by *RNASET2* which cleaves ssRNA within GU or AU motifs. In mammalian cells, it facilitates anti-pathogen immune response and is also implicated in cancer^35^ by responding to hydrogen peroxide, ultraviolet radiation, and inflammatory stimuli.^36^ LOF homozygous or heterozygous mutations, again within the protein-coding region of *RNASET2*, cause autosomal recessive cystic leukoencephalopathy which shows similar phenotypes to that of AGS disorder. The causal mechanism of the disease is attributed to the accumulation of uncleaved RNA substrates like rRNA molecules in lysosomes. This is in line with one of the main functional aspects of the enzyme in which it degrades exogenous or endogenous lysosomal RNAs, like rRNAs, and therefore is an important factor in the ribosome recycling pathway.^35^^,^^37^ It remains an open question to what extent the pathomechanism of the disease is determined by each of the two phenomena: disturbances in RNA turnover and the toxic effect of accumulated RNA. Organisms harboring *RNASET2* homozygous null mutations most often manifest stunted embryonic development that reduces lifespan.^38^

Despite the comprehensive study of the functions of the selected RNases and their mutation-associated diseases, an obvious reason for the nervous system's susceptibility to the mutations of these particular enzymes does not seem to emerge. Therefore, the next section provides an overview of the many commonalities between the enlisted diseases and their casual mutations, such as onset, immunological response, and disruption of de-centralized translation in neurons. Observing these signatures in patients with novel and unstudied mutations may indicate a phenotypic neurological vulnerability.

Due to the varied range of effects of heterozygous LOF mutations on enzymatic function (in turn affecting the neurons in this case), the resultant neurological disorders could either be early onset (*in utero* or postnatal) or late onset (adulthood). RNase H2, Ago-2, and RNase T2 mutations are associated with early-onset disorders. These diseases generally lead to non-fatal brain damage and the disease progression diminishes with age for some of them. In AGS caused by RNase H2 mutations, nuclear RNase H1 cannot replace the H2 enzyme in patients, but *in vitro* overexpression of RNase H1 in H2^−/−^ cells can partially compensate for enzyme function, particularly in the promotion of long interspersed nuclear element-1 (LINE-1) retrotransposition. In fact, accumulation of LINE-1 RNA:cDNA hybrids could be a source of immunostimulatory nucleic acids detected in AGS patients.^39^ Therefore, it can be hypothesized that the disease recession in the later years of life might be due to the partial supplementary role of RNase H1. On the other hand, angiogenin mutations causing ALS represent the sole late-onset neurodegenerative disorder within this group, characterized by progressive motor neuron loss. Several compensatory mechanisms functionally preserve neuron activity until approximately 50% are lost, explaining the disease's delayed onset.^40^^,^^41^ It can be implied that these compensatory mechanisms are additional stress–responsive pathways that get activated in the absence of angiogenin.

Next, in three out of the four mentioned neurological diseases, associated mutations lead to increased immunological responses in the central nervous system. In AGS, elevated type-I interferons trigger an innate immune response due to the accumulation of DNA damage by-products in the absence of functional RNase H2.^42^^,^^43^ Similarly, cystic leukoencephalopathy, caused by LOF RNase T2 mutations, exhibits an increased immune response like that observed in AGS. In fact, both these diseases phenotypically mimic human cytomegalovirus infection. This similarity can be reasoned out by the fact that alike viral infections, mutations in both enzymes lead to the accumulation of endogenous single-stranded nucleic acid molecules that are recognized by immune machinery.^42^^,^^44^ In familial cases of ALS caused by angiogenin mutations, there are reports about severe inflammatory reactions mediated by innate immunity cells like macrophages and mast cells.^45^ Hence, it can be stated that elevated immune response takes place because of the accumulation of undigested substrates present in the cell in the absence of a functional RNase.

Neuronal processes like axons and dendrites, being distant from cell bodies, rely on asymmetric proteome distribution to maintain cellular health and dynamic functionality. This asymmetry is achieved through localized translation or concentrated protein import.^46^ In RNase mutation-associated neurological diseases, disruption of decentralized translation pathways in neurons is evident. *AGO2* mutations hinder the regulation of local translation by reducing free Ago-2 protein availability in the cytoplasm, essential for translational regulation.^46^ Angiogenin mutations in ALS disrupt the 5′-tiRNA-triggered stress granule formation, involved in stress-responsive translation inhibition. Impeding stress granule formation can debilitate the neuroprotective role of angiogenin by impacting localized stress-induced translation.^47^ RNase T2 is involved in mitochondrial rRNA degradation. This has a positive correlation with nuclear and mitochondrial transcription, hence modulating ribosome biogenesis and translation. Thus, its disruption may interfere with localized protein synthesis.^48^ Collectively, disruption of asymmetric neuronal translation is a common theme in neurological disease pathogenesis, impairing synaptic plasticity.

## Growth-related disorders

RNase mutations that alter normal embryonic development and growth affect multiple organ and tissue systems at the same time and very often predispose the affected patients to various forms of cancerous and non-cancerous overgrowths. There are four human RNases, mutations in which are associated with abnormal growth disorders, namely, Dicer, DIS3-like exonuclease 2 (Dis3l2), zinc phosphodiesterase ELAC protein 2 (Elac2), and mitochondrial ribonuclease P catalytic subunit (MRPP3).

Dicer exhibits a tissue-wide expression pattern and plays a key role in normal organ development.^49^ In humans, it is encoded by the *DICER1* gene on chromosome 14. It primarily processes dsRNA or pre-miRNA molecules, cleaving them at specific sites to produce mature siRNA or miRNA molecules typically 20–24 nt in length. These molecules guide target binding within the RISC complex, contributing to RNA silencing (mRNA degradation or translational inhibition).^50^ Dicer generates mature miRNAs by cleaving the 5′ and/or 3′ arms of premature miRNA (pre-miRNA) duplex, giving rise to miR-5p and miR-3p.^51^ One of them occurs at higher concentrations in the cell than the other, and this ratio has a functional significance. Homozygous *DICER1* mutations are lethal to cells. Heterozygous LOF mutations in the RNase IIIb domain cause GLOW syndrome with unknown inheritance patterns (global developmental delay, lung cysts overgrowth, and Wilms tumor), disrupting the cellular 5p/3p miRNA ratio.^52^ The autosomal dominant pleuropulmonary blastoma (PPB) is particularly significant among all other *DICER1*-associated neoplastic conditions, such as multinodular goiter with or without Sertoli-Leydig cell tumors, and embryonal rhabdomyosarcoma stage 2. Approximately 70% of the total number of children with PPB carry *DICER1* germline mutations. PPB stands out as the most common and aggressive childhood lung malignancy.^53^ Mutations in both cases map to the protein-coding region of the *DICER1* gene. In fact, GLOW syndrome-specific mutations are exclusively located at the RNase IIb domain coding region.

Dis3l2, encoded by the *DIS3L2* gene on chromosome 2, is part of the DIS3 group of proteins. Unlike Dis3 and Dis3l1, which are components of the RNA exosome complex involved in mRNA degradation, Dis3l2 acts independently of the exosome. It mediates RNA degradation of coding and non-coding substrates, regulated by the addition of non-template 3′-uridine residues by TUTases.^54^ Homozygous and/or heterozygous mutations in *DIS3L2* cause the autosomal recessive Perlman syndrome, an overgrowth disease with symptoms including organomegaly, hypotonia, and renal abnormalities.^55^ Most of these mutations lead to erroneous transcription, like complete deletion of one or more exons, truncated transcript formation, splice site mutations, and complete absence of the transcript or mRNA instability.^55^ In LOF mutations associated with Perlman syndrome, there is a notable accumulation of aberrant 3′-uridylated 7SL ncRNA which is a component of the signal recognition particle (SRP) and is involved in ER-translation regulation.^56^ The accumulation of uridylated forms of 7SL ncRNA disrupts ER-targeted translation and calcium homeostasis.^57^^,^^58^ However, several mechanistic details of the disease remain to be elaborated, including the role, if any, of the affected pool of tRNA, rRNA, and miRNA.

Finally, zinc phosphodiesterase ELAC protein 2 (Elac2) encoded by *ELAC2*, and mitochondrial ribonuclease P catalytic subunit (MRPP3) encoded by protein-only RNase P catalytic subunit (*PRORP*) are the catalytic effectors of the mitochondrial pre-tRNA (mt-pre-tRNA) 3′- and 5′-end processing pathways, respectively. While Elac2 itself functions as an enzyme, MRPP3 serves as the catalytic subunit of the RNA-protein complex RNase P. Together, they catalyze the processing of mitochondrial polycistronic transcripts, including mt-tRNAs, mRNAs, and rRNAs, into mature molecules.^59^ The absence of functional forms of MRPP3 and Elac2 causes autosomal recessive mitochondrial diseases like combined oxidative phosphorylation deficiency COXPD-54 and -17, in that order.^60^^,^^61^ Additionally, *ELAC2* mutations might also be associated with hereditary prostate cancer 2 (HPC2). Causal homozygous or compound heterozygous mutations for both diseases are predominantly located within the protein-coding regions of their respective genes.^59^^,^^62^ The manifestations of COXPD-54 are pleiotropic and include sensorineural hearing loss, primary ovarian insufficiency, developmental delay, and leukoencephalopathy.^62^ In contrast, the major symptom of COXPD-17 is hypertrophic cardiomyopathy.^59^ The molecular mechanism of both disorders is attributed to the impairment of mitochondrial translation. As proteins instrumental in ATP production are exclusively encoded by mtDNA, disrupted translation in these organelles abolishes the formation of functional oxidative phosphorylation (OXPHOS) components. However, it should be noted that the phenotypes of both diseases are clearly different. Moreover, Elac2 has a nuclear form, in addition to the mitochondrial one, and is involved in the processing of nuclear ncRNAs.^63^ Further research is required to gain a complete understanding of the pathway initiated by the alteration of RNase function ultimately leading to such diverse phenotypes.

The multiorgan phenotypic manifestations are the most consistent and intriguing trend among the mentioned growth abnormalities. Consequently, we delved deeper into the molecular mechanisms and targets of the relevant RNases to identify common traits across these diseases, namely, mutated protein domain(s), molecular targets, and phenotypic manifestations. Studying these factors in detail using suitable model organisms could guide future research and therapeutic strategies.

Disease-causing mutations in this group are all located in the catalytic domain of each enzyme. Mutations in *DICER1* that are concentrated only within one of the five key codons of the metal-binding cleft of RNase IIIb ribonuclease domain culminate into GLOW syndrome. This domain specifically cleaves 5p-miRNA molecules which are in fact depleted in the disease. However, the unaffected and still functional RNase IIIa domain specific for 3p-miRNA cleavage may be a cause for the signature increase in accumulation of the 3p molecules.^52^^,^^64^ Mutations in the C-terminal catalytic metal-binding metallo-β-lactamase (MBL) domain of Elac2 hinder its 3′-pre-tRNA processing RNase Z activity, resulting in the development of COXPD-17.^65^ Similarly, LOF mutations in the metallonuclease domain of the only ribonucleolytic subunit of mt-RNase P cause yet another mitochondrial OXPHOS deficiency, COXPD-54.^62^ Lastly, proteins from most of the mutated DIS3L2 transcripts in Perlman syndrome are predicted to abolish the enzyme's RNA binding domain, resulting in loss of enzymatic function. This RNA binding domain serves as the catalytic domain for the entire ribonuclease II enzyme family, which includes Dis3l2.^55^

All enzymes in the group target regulatory small ncRNAs, and mutations causing inefficient cleavage of these targets lead to deregulated translation machineries. Domain-specific DICER1 mutations in GLOW syndrome cause an increased accumulation of various 3p-miRNA molecules, antagonistically targeting key positive effectors of the phosphoinositide 3-kinase (PI3K)-protein kinase B (AKT)-mammalian target of rapamycin (mTOR)-mediated growth signaling pathway. For instance, the 3p-miRNA molecules negatively regulate mTOR inhibitors like TSC complex subunit 1 (TSC1) and positively regulate the downstream pro-proliferative driver, ribosomal protein S6 kinase (S6K).^52^ Protein products of both *PRORP* and *ELAC2* genes are involved in the maturation process of mt-tRNAs. Therefore, mutations in both genes lead to an anticipated mitochondrial translation disruption. As gene products instrumental in mitochondrial ATP production are exclusively encoded by mtDNA, the translation of several OXPHOS-related mRNAs is disrupted.^59^, ^60^, ^61^, ^62^ Dis3l2 targets non-coding RNA molecules of several types. Disease-causing homozygous/heterozygous mutations lead to the accumulation of abnormal 3′-uridylated 7SL ncRNA ultimately manifesting the associated molecular and phenotypic defects.^56^

The tissue-wide effects of these diseases can be traced back to specific mutations and their impact on major organelles. Recurrent defects in certain organs like kidneys and lungs owing to 5p- and 3p-miRNA imbalance in the case of mosaic *DICER1* mutations indicate that organ-specific miRNAs are vital for the functioning of these organs.^66^ Next, as mitochondria are the energy production unit of the cell, multiple organs are also frequently affected in case of mitochondrial disorders like COXPD-54 and -17.^59^, ^62^, ^67^ *DIS3L2* mutations linked to Perlman syndrome disrupt the proper development of multiple organs, especially those crucial for inter-organ and environmental interactions, such as the pancreas, nervous system, muscles, and kidneys.^55^^,^^58^

## Hematopoietic disorders

Mutations in genes encoding two very functionally unrelated RNases, namely poly(A)-specific ribonuclease (PARN) and Schlafen family member 14 (SLFN14), particularly target the hematopoietic system.

PARN, encoded by the *PARN* gene on chromosome 16, acts as a 3′-poly A tail deadenylase. Traditionally known for its role in mRNA degradation by removing adenine residues from 3′ tails, it has been recently revealed to be involved in the processing and stabilization of various small ncRNA classes. In certain ncRNAs like hTR, rRNA, Y RNAs, H/ACA box snoRNAs, and scaRNAs, the addition of a 3′-poly A tail acts as a degradation signal, and PARN counteracts degradation by removing these tails.^68^, ^69^, ^70^ In diseases associated with *PARN* mutations, the stability of H/ACA box snoRNAs and telomerase RNA component (TERC) is crucial. The protein mediates the 3′-end maturation of both H/ACA box snoRNA and TERC, despite the latter lacking a 3′-poly A tail. Subsequently, it was found that TERC has a 3′- H/ACA box snoRNA motif which is in fact recognized by the enzyme for its activity.^71^ TERC stability is vital for telomerase activity at DNA ends. Bi- or mono-allelic mutations in *PARN* lead to impaired telomere replication and abnormal DNA damage response, causing autosomal recessive dyskeratosis congenita-6 (DKCB6), characterized by bone marrow failure, and the autosomal dominant disorder, pulmonary fibrosis and/or bone marrow failure syndrome, telomere-related, 4 (PFBMFT4). Some mutations in the protein-coding region of the gene lead to changes like the substitution of key amino acid residues.^71^ Both diseases, DKCB6 and PFBMFT4, are associated with bone marrow failure. This can be reasoned out by the fact that maintenance of telomere length is important for highly proliferative cells like stem and progenitor cells to promote tissue renewal and longevity. Such cells harboring *PARN* mutations undergo premature senescence and death due to telomere shortening, particularly affecting tissues with high replicative turnover like the bone marrow and lung.^72^^,^^73^ Although several lines of evidence directly link the diseases caused by *PARN* mutations with telomere instability, the question arises as to how much disturbances in the biogenesis of several other key classes of ncRNAs contribute to the development and course of the disorders.

SLFN14, encoded by the *SLFN14* gene on chromosome 17, is an endoribonuclease that binds ribosomes to cleave rRNA and ribosome-bound mRNA and thereby degrades ribosomal subunits and represses translation. Homozygous or heterozygous missense mutations in the protein-coding region of *SLFN14* affect the AAA domain of the protein and phenotypically cause an autosomal dominant disorder, platelet-type bleeding disorder-20, with thrombocytopenia due to dysregulated ribosomal homeostasis that is critical for anucleate platelet and reticulocyte formation.^74^^,^^75^ This AAA domain is predicted to bind to ATP/GTP molecules during nucleic acid metabolism thereby enabling enzyme function.^76^ Despite mutations, the protein still retains ribosome binding and rRNA cleavage activity. However, there is a reduced expression of the wild-type form, which is caused due to the dominant-negative effect of the mutation. The mutants form heterooligomers with the wild-type counterparts leading to a partially unfolded form that undergoes degradation by the cellular unfolded protein response pathways. In the context of platelet biogenesis, mature platelets and red blood cells are generally devoid of RNA. From the current knowledge about the protein's function in rRNA degradation, a role of the enzyme in RNA clearance during megakaryocyte and reticulocyte maturation can be hypothesized.^77^

## Other disorders

Mendelian disorders caused due to mutations in some RNases like ribonuclease H1, 2-5A-dependent ribonuclease, and poly(A)-specific ribonuclease PNLDC1 cannot be categorized into any of the above groups due to heterogeneity in the type of tissue that is affected.

DNA:RNA hybrid targeting enzyme ribonuclease H1 (RNase H1), encoded by the *RNASEH1* gene on chromosome 2, is targeted to the nucleus and mitochondria using alternative translation initiation sites.^78^ In the nucleus, it is functional in R-loop removal, processing Okazaki fragments, DNA repair, and telomere elongation, and the mitochondrial activities revolve around mtDNA replication and transcription. Nevertheless, the functional mitochondrial isoform seems to be indispensable, as *RNASEH1*^−/−^ mutants are embryonic lethal due to severe dysfunction of the mitochondria. Heterozygous *RNASEH1* mutations in the catalytic domain coding region cause autosomal recessive progressive external ophthalmoplegia with mitochondrial DNA deletions-2, commonly referred to as chronic progressive external ophthalmoplegia (CPEO), which is phenotypically presented mainly with muscular weakness, overall muscular dysfunction, dysphagia, and spinocerebellar manifestations. Most CPEO-causing mutations in *RNASEH1* identified so far map to the catalytic domain.^79^ The mechanistic background of the disease is linked to defects in mtDNA replication, which leads to the depletion and deletion of mtDNA. This further results in a deficiency in OXPHOS proteins and disrupted ATP production.^80^ As muscle cells have very high energy demands, the inefficient mitochondrial energy production pathway primarily affects the muscle tissue.^81^ It is believed that nuclear deficiency of RNase H1 can be compensated by RNase H2, which explains why mitochondria are exclusively affected by *RNASEH1* mutation.^79^ Nevertheless, why the phenotype of the disease varies from mild to more severe remains unclear.

*RNASEL* encodes 2-5A-dependent ribonuclease, an enzyme regulated by antiviral interferon signaling. Interferon cytokine signaling induces the expression of 2′,5′-oligoadenylate synthetase (OAS) genes. Once activated by dsRNA molecules, the OAS enzyme generates 2′-5′-linked oligoadenylate molecules (2–5A) from ATP, promoting RNase L dimerization and activation. RNase L then cleaves viral and cellular ssRNA, inhibiting viral replication and inducing host cell apoptosis.^82^ Additionally, recent studies have unveiled its roles in regulating proliferation, apoptosis, differentiation, and autophagy. These functions are mediated by translational control mechanisms like rRNA or mRNA cleavage or premature translation termination.^83^^,^^84^ The enzyme's pro-apoptotic functions are especially implicated in its tumor suppressive roles. As a result, *RNASEL* deficiencies are implicated in several cancer types, most prominently in autosomal dominant hereditary prostate cancer 1 caused by *RNASEL* homozygous or heterozygous LOF mutations.^83^^,^^85^ A number of disease-causing mutations affect the translation by either mutilating the start codon to abolish translation or by adding stop codons at various premature positions. Some mutations cause the loss of the wild-type allele.^86^

Poly(A)-specific ribonuclease PNLDC1 (Pnldc1) encoded by *PNLDC1*, is a deadenylase homologous to PARN that coexists alongside PARN in most lower eukaryotes and vertebrates.^87^ It was first functionally characterized in silkworms where it cleaves 3′ tails of pre-piRNAs to produce mature piRNAs.^89^ In mammals, mature piRNAs in complex with PIWI proteins maintain germ cell development, fertility, and genome integrity via the piRNA-guided RNA silencing pathway. In line with that, the enzyme is specifically expressed in the testes, namely, in meiotic spermatocytes and embryonic stem cells. Also, its expression is tightly regulated by promoter methylation during development, whereby it is abundantly expressed early in the process, but later in differentiation, the expression gradually diminishes. Cumulatively, it can be concluded that *PNLDC1* is expressed in undifferentiated cell types where it is implicated in lineage re-programming. Compound heterozygous or homozygous mutations in *PNLDC1* cause autosomal recessive spermatogenic failure 57, which manifests with male infertility due to error-prone meiosis and spermatogenic arrest. Most mutations map to the evolutionarily conserved CAF exonuclease domain of the protein.^12^ The disorder is caused by the accumulation of pre-piRNA with unprocessed 3′ ends and the de-repression of specific transposons like LINE-1 in spermatocytes.^88^^,^^89^ Notably, it was found that Pnldc1 acts as a poly(A)-specific exonuclease *in vitro* and further studies in this direction are required to reveal whether this activity is important for poly(A) RNA processing in germ cells.^88^

## Insights from model organisms

Although much has been learned about Mendelian genetic diseases caused by mutations in RNase genes, many questions remain. They focus on how mutations connect to molecular phenotypes and impact the metabolism of all substrate classes for a given enzyme. Answering these questions is crucial for the development of biomarker-based diagnostic methods, and, at least in some cases, also for the design of therapeutic approaches. Studying human patients to understand Mendelian diseases linked to RNases provides limited insights due to ethical and technical constraints. Although human cell lines offer a broader avenue for investigation, they may not fully capture the intricate *in vivo* dynamics of these enzymes. Given the evolutionary conservation of RNases, model organisms are invaluable for understanding the genetic and molecular basis of diseases.^90^ Mutations in genes shared between humans and model organisms can lead to similar phenotypes, providing insights into the disease mechanisms and potential therapeutic targets.^91^ Moreover, model organisms allow controlled studies of gene knockouts or mutations, helping researchers to decipher the roles of specific genes and pathways in disease progression.^92^ Rodents with their close evolutionary relatedness and well-characterized biology offer the best model for human diseases. However, for certain disorders, simpler models are also favored, facilitating research using limited resources and time. This enables the rapid generation of molecular and phenotypic insights that can lead to advancements in diagnosis and therapy development. For instance, while worms and flies are evolutionarily distant from humans, approximately 83% and 85% of the ∼4000 genes associated with Mendelian diseases listed in OMIM have homologs in *C. elegans* and *D. melanogaster*, respectively. Their high fecundity and suitability for molecular studies make them efficient for characterizing disease-associated variants. For phenotypes involving vertebrate-specific organs, tissues, and cells, the fish model *D. rerio* offers a faster, cost-effective alternative.^7^

Discoveries from animal model studies, rather than cell lines, often revealed the molecular basis of diseases. Notably, even non-mammalian models, typically considered less applicable, have contributed significantly in this regard. Examples include the functional characterization of Pnldc1 in the silkworm^93^ or the discovery of RNA accumulation in lysosomes during *RNASET2* disruption in zebrafish.^112^ Despite limitations due to evident physiological differences, which can restrict the direct translation of findings to humans,^94^ animal models complement studies in human subjects and cell lines, offering a broader understanding of disease biology.

Studying homologous genes shared between two species, inherited from a common ancestor, presents a powerful means for understanding gene function and evolution. Of particular interest are orthologous genes, which diverge following a speciation event yet maintain their principal function, offering insights into how conserved are the genetic mechanisms that underpin specific biological processes and diseases.^90^ Also, for human RNases associated with Mendelian diseases, there are data obtained in animal model studies that may contribute to a better understanding of the pathomechanisms.

## Phylogenetic analysis

To provide cross-species insight into the fundamental biological functions of human RNases and their role in Mendelian diseases, we conducted a phylogenetic analysis using a variety of model organisms. Trusted orthologs of these enzymes were retrieved from comprehensive databases like the Alliance of Genome Resources.^95^ This selection includes species such as mouse (*Mus musculus*), rat (*Rattus norvegicus*), frog (*Xenopus tropicalis*), zebrafish (*D. rerio*), representing phylum *Chordata*, fruit fly (*D. melanogaster*, phylum *Arthropoda*), nematode (*C. elegans*, phylum *Nematoda*), and yeast (*Saccharomyces cerevisiae*, phylum *Ascomycota*), reflecting the broad phylogenetic distribution and importance of these enzymes. We focused on RNases implicated in Mendelian diseases, having homologs in at least five of the eight selected model organisms, namely, RNase H2, Ago-2, RNase T2, Dicer, Dis3l21, Elac2, MRPP3, PARN, and RNase H1. The analysis focused on specific protein sequences selected for their relevance and orthology. Notably, the selection of RNase T2 and Ago-2 sequences required a tailored approach. The mouse genome contains two orthologs of the human *RNASET2* gene, termed *Rnaset2a* and *Rnaset2b*, both of which are identical at the protein level^96^; Rnaset2a protein sequence was used in this analysis. It is also worth mentioning that *D. melanogaster* Ago-1, unlike Ago-2, shares functional similarities with human Ago-2 and is recognized as a trusted ortholog to human Ago-2.^97^ Consequently, the fly Ago-1 protein sequence was also chosen for alignment. Similarly, *C. elegans*^98^, ^99^, ^100^ has two genes, *alg-1* and *alg-2*, both orthologous to human *AGO2* and *AGO1*, sharing evolutionary ancestry and functional similarities with both human Argonaute proteins. For this analysis, we used the protein sequence encoded by the *alg-1* gene. To enhance the understanding and accessibility of the comparative analysis of RNases across selected species, we have compiled a comprehensive dataset. This dataset, presented in Table S1, includes detailed information about the effects of RNase changes and disease correlations across species.

**Figure 2:**
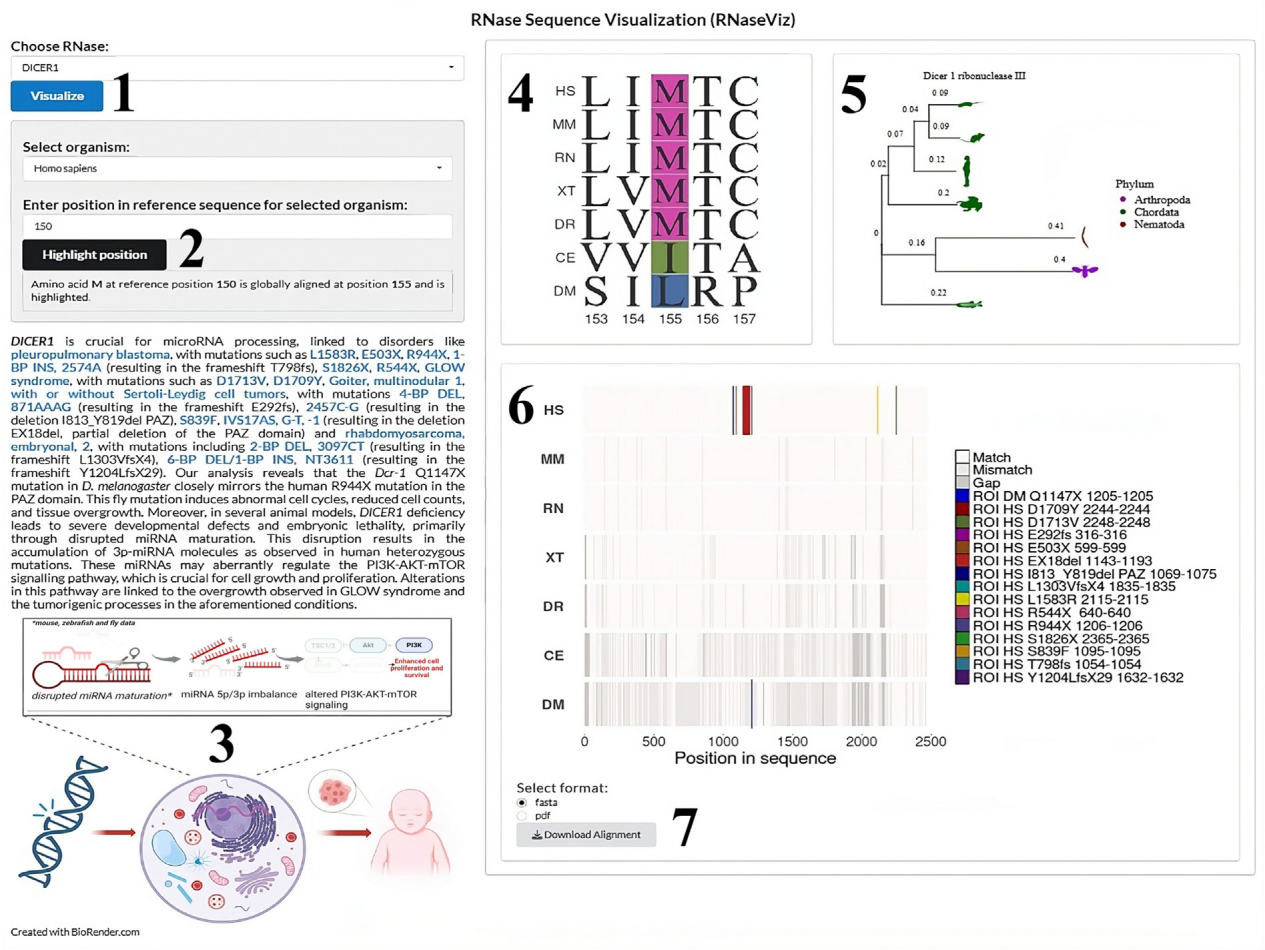
Interactive analysis of RNase phylogeny and sequence conservation across species (RNaseViz web tool). Users can select a specific RNase for analysis from a list of available sequences (1) and input a residue position based on the reference sequence in the selected organism (2), which will be highlighted in the alignment. Detailed information about the chosen RNase and the potential molecular consequences of mutations is provided (3). The sequence alignment highlights the specified residue along with two residues on either side (4) and an interactive phylogenetic tree displays the evolutionary relationships between the selected RNase sequences (5). The selected RNase sequence is aligned across eight different species, with the human sequence used as the reference. Users can highlight each residue (6). Global sequence alignments can be downloaded in FASTA or PDF formats (7).

We conducted a phylogenetic analysis of selected RNases in model organisms to elucidate the evolutionary relationships. The phylogenetic trees were generated based on amino acid sequences using the ClustalOmega tool within the msa package in R for sequence alignment and for neighbor-joining tree estimation, and the ggtree package for visualization of the trees.^101^, ^102^, ^103^, ^104^ To enhance the visualization and analysis of RNase sequences and their disease-associated mutations, a novel web tool, RNaseViz https://norreanea.shinyapps.io/RNaseViz/, was developed in R Shiny.^105^ It allows users to select specific RNases, visualize their sequence conservation across species through multiple sequence alignments, explore interactive phylogenetic trees, and illustrate the potential molecular effects of RNase mutations (Fig. 2). Additionally, users can download global sequence alignments in FASTA format or enriched PDF reports that include human mutations listed in Table S1, color-coded based on their associated diseases, for offline analysis. The source code and further details about the tool can be accessed on GitHub (https://github.com/Norreanea/RNaseViz-RNase-Sequence-Visualization-Tool).

**Figure 3:**
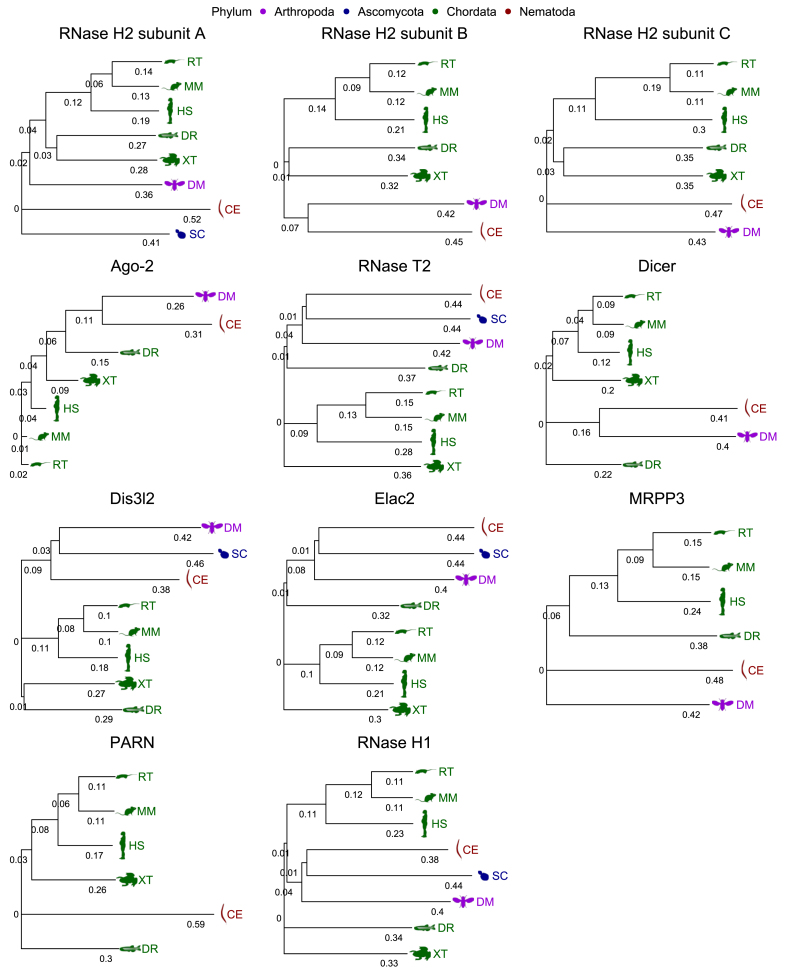
Phylogenetic analysis of ribonucleases across diverse eukaryotic lineages. The length of the branches indicates the evolutionary distance between sequences (longer branches indicate greater distance). The positioning of the nodes shows the branching patterns and suggests common ancestors for groups of sequences.

The RNase H2 subunit A tree clusters all chordates together, with separate branches for *D. melanogaster*, *C. elegans*, and *S. cerevisiae*, indicating distinct evolutionary paths (Fig. 3). In the RNase H2 subunit B tree, chordates are divided into two clusters: one for non-mammalian vertebrates (*D. rerio* and *X. tropicalis*) and another for mammals (*H. sapiens*, *M. musculus* and *R. norvegicus*). Notably, *C. elegans* (*Nematoda*) and *D. melanogaster* (*Arthropoda*) form a single cluster, reflecting their close relationship within the *Ecdysozoa* clade. The RNase H2 subunit C tree mirrors the pattern in subunit B but separates *C. elegans* and *D. melanogaster*. In the Ago-2 tree, *C. elegans* and *D. melanogaster* share a common ancestor, and vertebrates do not form a homogenous cluster. The RNase T2 tree shows *C. elegans* and *S. cerevisiae* clustering together, possibly due to convergent evolution or horizontal gene transfer. In the Dicer tree, all chordates, except *D. rerio*, are grouped together, while *C. elegans* and *D. melanogaster* stem from a distinct node. The Dis3l2 tree places *D. melanogaster* and *C. elegans* close to *S. cerevisiae*, with mammalian and non-mammalian vertebrates forming separate clusters, which suggests a fundamental and conserved role for Dis3l2. In the Elac2 tree, mammals cluster together, while *X. tropicalis* and *D. rerio* are placed on individual branches, with *D. rerio* notably closer to *Arthropoda*, *Nematoda*, and *Ascomycota*. Similarly, in the MRPP3 tree, chordates are grouped together, with *C. elegans* and *D. melanogaster* appearing on separate branches. In the PARN tree, *D. rerio* is divergent within vertebrates, and the other chordates form a cluster, while *C. elegans* is positioned on a separate branch, underscoring a distinct evolutionary trajectory of this gene in nematodes. Lastly, for RNase H1, mammals form a cluster, and the other chordates, *D. rerio* and *X. tropicalis*, are separated. *D. melanogaster*, *C. elegans*, and *S. cerevisiae* cluster together.

In general, as expected, chordate species tend to cluster together, in line with a close evolutionary relationship. Arthropods tend to be quite distinct across all RNases, which reflects a significant evolutionary divergence from the other phyla represented here. *Ascomycota* and *Nematoda* vary in their placement but often are distinct from chordates, reflecting their evolutionary distance.

The distance matrix, based on amino acid sequences, complements the phylogenetic trees by quantifying the evolutionary distances between the RNases of different species and humans (Table 2). It adds a quantitative measure to the qualitative insights from the phylogenetic trees (distance values indicate the genetic differences between the RNase sequences of different species and humans; the lower the value, the greater the similarity). Identifying species with high similarity in RNase sequences to humans helps in selecting appropriate model organisms for experimental studies. Taking this analysis as a starting point, we present below the information on human Mendelian diseases gained from ortholog analyses.

**Table 2:** Comparative distance matrix of ribonucleases across species relative to *H. sapiens*.

	RNase H1	RNase H2A	RNae H2B	RNase H2C	Ago-2	Dicer	Elac2	RNase T2	Dis3l2	PARN	MRPP3
*M. musculus*	**0.46**	**0.37**	**0.43**	0.59	**0.08**	**0.26**	**0.42**	0.56	**0.36**	**0.33**	**0.47**
*R. norvegicus*	**0.47**	**0.39**	**0.43**	0.59	**0.09**	**0.25**	**0.42**	0.56	**0.35**	**0.34**	**0.49**
*X. tropicalis*	0.67	0.62	0.67	0.78	**0.18**	**0.40**	0.61	0.72	0.56	0.50	
*D. rerio*	0.70	0.61	0.72	0.79	**0.29**	**0.43**	0.64	0.75	0.60	0.57	0.75
*D. melanogaster*	0.78	0.72	0.83	0.85	0.51	0.78	0.80	0.84	0.82		0.85
*C. elegans*	0.77	0.90	0.87	0.88	0.56	0.78	0.86	0.86	0.76	0.88	0.91
*S. cerevisiae*	0.84	0.78					0.86	0.85	0.86		

Note: Empty cells indicate that a particular RNase sequence has not been deemed a trusted ortholog or selected species does not possess particular RNase genes. Values in the table less than 0.5 are in bold.

## A cross-species approach to understanding human diseases

Studying RNase function and dysfunction across species reveals how similar genetic alterations result in comparable disease states aiding in understanding molecular mechanisms and development of targeted therapies. To describe the effects of RNase mutations in model organisms, in the following sections, we refer to the residue positions in the global alignments available in RNaseViz.

**Figure 4:**
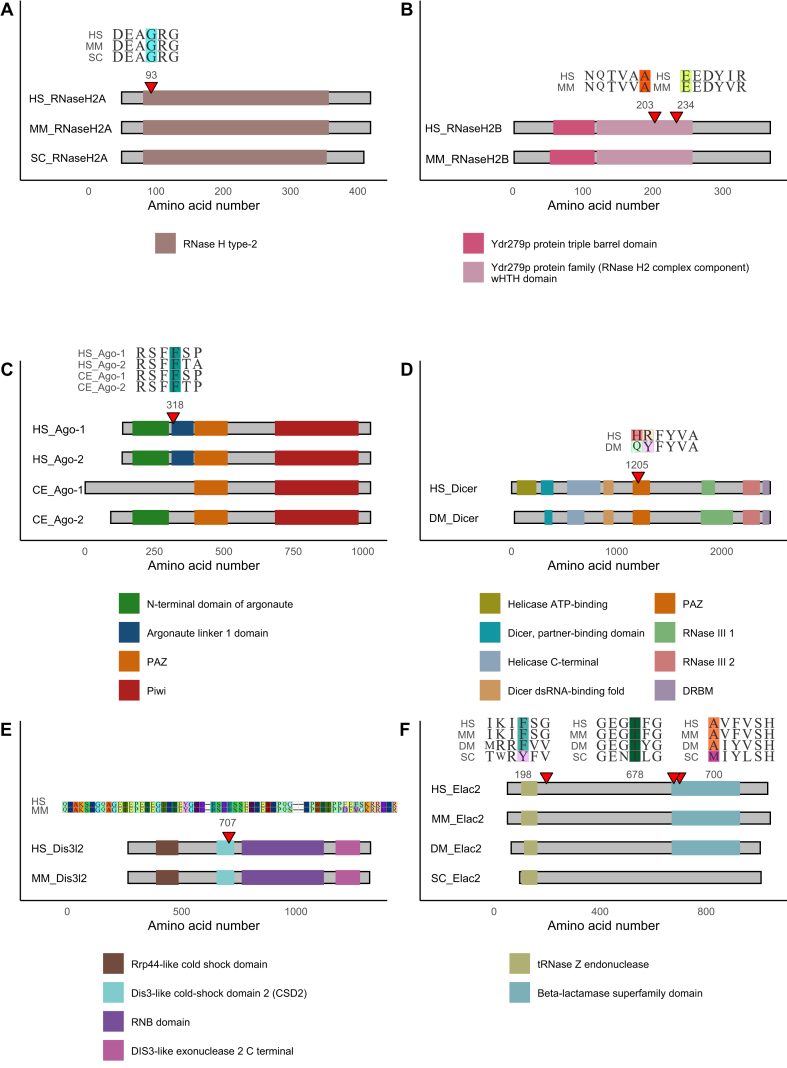
Comparative sequence analysis of ribonuclease mutations across species. The domain organization is illustrated exclusively for those RNases that exhibit disease-specific mutations, rather than gene knockouts. Amino acid sequence alignments highlight the mutation sites (color background and position number), flanked by adjacent residues (A–D, F) or deletion of a whole exon (E). Aligned protein sequences are depicted as gray rectangles with black borders, with particular domains shown as color rectangles according to the legend, and positions indicated on the axis. Mutation sites are indicated by red triangles. (A) RNase H2A; (B) RNase H2B; (C) Argonaute; (D) Dicer; (E) DIS3-like exonuclease 2 (Dis3l2); (F) Elac2.

Amino acid substitutions in RNase H2A, RNase H2B, and RNase H2C across various species underscore the critical role of the RNase H2 complex in maintaining genomic stability, regulating immune responses, and controlling developmental processes. In mouse models, the RNase H2A G37S (corresponding to human RNase H2A G37S, globally aligned at position 93, see RNaseViz) mutation leads to perinatal lethality and activation of the innate immune system via the cGAS-STING pathway, caused by the enzyme's inability to effectively remove misincorporated ribonucleotides and RNA:DNA hybrids^106^ (Fig. 4A). This failure initiates a DNA damage response and cellular senescence. Similarly, yeast cells harboring the corresponding G42S mutation exhibit increased ribonucleotide incorporation into the nuclear genome.^8^^,^^18^^,^^107^^,^^108^ Additionally, mouse models with the RNase H2B A174T mutation, corresponding to human RNase H2B A177T (globally aligned at position 203), do not show an immunological abnormality, whereas RNase H2B E202X (globally aligned at position 234) mutants display impaired proliferation and accumulation in the G2/M phase of the cell cycle^109^ (Fig. 4B). This condition results from chronic DNA damage response activation, marked by increased single-strand breaks, elevated histone H2AX phosphorylation, and activation of p53 target genes, such as the cyclin-dependent kinase inhibitor 1 (*p21*). Deficiency in RNase H2B function leads to excessive ribonucleotide incorporation into DNA, triggering spontaneous DNA breaks and consequent DNA damage responses. Furthermore, *Rnaseh2c* knockout in mice causes severe DNA damage from ribonucleotide misincorporation, leading to an extensive DNA damage response and significant developmental anomalies resulting in early embryonic death.^21^ Although the RNase H2C ortholog for the yeast model has not been designated as a trusted sequence by the Alliance for Genome Resources, the yeast RNase H2C K46W mutation (corresponding to human RNase H2C R69W) increases frequencies of cytosine and guanine ribonucleotides (rC and rG), particularly rCMP, at specific genomic sites.^18^ In AGS, DNA damage due to RNase H2 dysfunction leads to neuroinflammation and chronic activation of neuroimmune pathways, as demonstrated by the mouse models.^110^

*AGO2* mutations in humans are linked to a broad spectrum of developmental and neurological abnormalities due to impaired RISC formation or increased binding affinity of Ago-2 to mRNA targets.^32^ Correspondingly, in the nematode model, mutations or knockdown of *alg-1* and *alg-2* lead to embryonic developmental arrests and specific defects in vulval formation and hypodermal cell differentiation, along with significant disruption of miRNA biogenesis.^98^, ^99^, ^100^ The vulnerability of *alg-2* knockout worms to oxidative stress or heat shock reflects similar sensitivity to environmental and physiological stressors in humans with LESKRES. In particular, the F180del and the G199S amino acid substitution in *C. elegans* Ago-1 are linked to significant disruptions of miRNA expression profiles. These residues, located in the linker 1 domain, are involved in the interaction with the miRNA and the target mRNA. Mutations here likely directly translate into the various aspects of miRNA activity dysregulation observed in LESKRES.^32^ Notably, the F180del mutation in *C. elegans* Ago-1 corresponds to the F180del mutation in human Ago-1, linked to neurodevelopmental disorder with language delay and behavioral abnormalities, with or without seizures, and the F182del mutation in human Ago-2. These mutations at conserved sites (globally aligned at position 318) highlight the functional parallels not only between *C. elegans* Ago-1 and human Ago-2 but also between human Ago-1 and Ago-2, while underscoring their roles in distinct disease contexts (Fig. 4C).

Studying *RNASET2* mutations offers another compelling example of how animal models can illuminate the pathogenesis of complex human diseases. In humans, these mutations are linked to leukoencephalopathy, and the resulting phenotypes are paralleled in worms, zebrafish, mice, and yeast.^38^^,^^96^^,^^111^, ^112^, ^113^ For instance, *C. elegans*^38^ *rnst-2* mutants and zebrafish^111^^,^^112^ *rnaset2* knockout show enlarged lysosomes and increased microglia activation, as well as systemic inflammation and brain-specific pathologies, such as neuroinflammation and cognitive impairment. As detailed above, mutations in *RNASET2* cause an accumulation of uncleaved rRNA leading to lysosomal dysfunction and storage disorders. This is observed in zebrafish knockouts and *C. elegans* with G119E and P55X substitutions. Similarly, the accumulation of rRNA disrupts cellular stress responses and contributes to leukoencephalopathy by activating innate immune pathways, including the up-regulation of *interferon-1* and interferon-stimulated genes in mouse models.^96^ This results in neuroinflammation and impaired phagocytosis by activated microglia. Additionally, *RNY1* deficiency in yeast^113^ leads to stabilized tRNA levels and prevents the formation of tRNA fragments, impairing oxidative stress responses and inhibiting apoptosis. The consistent observation of impaired autophagy and lysosomal dysfunction across these species highlights these processes as potential therapeutic targets. The inflammatory response, particularly the up-regulation of interferon-stimulated genes, suggests that modulating the immune response could be another strategy for mitigating disease symptoms, providing multiple targets for potential interventions in treating *RNASET2*-associated disorders.

Moving from the impact of miRNAs in neural development, our comparative analysis also delves into the Dicer protein sequence across species, focusing on its association with growth-related disorders. Specifically, we observed a proximity of the Dicer Q1147X substitution in *D. melanogaster* (globally aligned at position 1205) to the corresponding human Dicer R944X substitution (globally aligned at position 1206),^114^^,^^115^ both located in the PAZ domain (Fig. 4D). This offers a robust model for understanding conditions such as PPB. In flies, the Q1147X substitution results in abnormal mitotic cell cycles and reduced cell counts, mirroring the PPB phenotype. These flies also show developmental anomalies and tissue overgrowth, similar to the uncontrolled cell proliferation and tumorigenesis in PPB, supporting *D. melanogaster* as a model for disease study. The abnormal stress responses in mutant flies may reflect the increased vulnerability of PPB tissues to physiological stress, illustrating compromised resilience mechanisms due to *DICER1* mutations. Given the crucial role of Dicer in miRNA processing, examining how the Q1147X mutation influences neuroblast proliferation and differentiation in flies can shed light on the enzyme's function in human stem cell populations, especially in the lungs where PPB originates. In zebrafish^116^ and mouse,^117^^,^^118^ deficiencies in Dicer lead to severe developmental abnormalities and embryonic lethality, mainly due to disrupted miRNA maturation. This disruption often results in the accumulation of 3p-miRNA molecules, akin to observations in humans with heterozygous mutations. These miRNAs aberrantly regulate the PI3K-AKT-mTOR pathway, critical for cellular growth and proliferation. This highlights the potential for further investigations into how *DICER1* mutations can impact critical growth-regulating pathways.

The phenotypes in fly^119^ and mouse^120^^,^^121^ models carrying *DIS3L2* mutations closely align with the characteristics of Perlman syndrome, particularly regarding overgrowth and developmental challenges.^55^ These mutations include the fly's Dis3l2 V7GfsX10 and deletions of exons 10 and 11 in mice, corresponding to deletions in exons 9 and 10 in the human *DIS3L2* gene (Fig. 4E). Furthermore, the failed degradation of 3′-uridylated-poly(A) mRNAs in these models leads to up-regulation of the oncogene *Igf2*, which promotes tumorigenesis. The targeted deletion of *Dis3l2* in mouse oocytes has elucidated the gene's broader biological implications, suggesting its impact on fertility could be relevant to the condition, and opening potential pathways for targeted treatments.

Exploring OXPHOS deficiency, the conserved nature of mitochondria across species provides a platform to understand how mutations in *ELAC2* and *PRORP* affect mitochondrial RNA processing, translation, and respiratory chain function. This investigation illuminates the underlying mechanisms of oxidative phosphorylation deficiencies. Specifically, substitutions such as the Elac2 T513I (globally aligned at position 678) in yeast^59^ and T494I (globally aligned at position 678) in flies, both corresponding to human T520I, as well as F155L (globally aligned at position 198) in flies,^122^ corresponding to human F154L, lead to impaired mitochondrial function and energy production, suggesting potential treatments that could enhance mitochondrial RNA processing or correct defective protein synthesis in disorders like COXPD17 might be first tested in these models^59^^,^^122^ (Fig. 4F). In mice,^63^ *Elac2* knockout is lethal in homozygous embryos and leads to dilated cardiomyopathy and premature death in heart and skeletal muscle-specific knockouts due to disrupted RNA processing, production of unprocessed mitochondrial transcripts and a novel class of tRNA fragments, impaired protein synthesis, and gene expression. Furthermore, mouse^123^ models reveal that substitutions in Elac2, including the A537T (corresponding to human A541T, globally aligned at position 700) and prostate-specific A537T substitutions (corresponding to mutations linked with HPC2 in humans), promote cellular proliferation and tumorigenesis. These mutations lead to the accumulation of unprocessed mt-tRNA molecules, disrupting mitochondrial protein synthesis and respiration, and consequently decreasing OXPHOS complex levels. This results in reduced ATP production and elevated levels of reactive oxygen species, contributing to mitochondrial disorders by impacting energy production and inducing cellular stress.^124^ In conditions like HPC2, these disruptions impair both mitochondrial and nuclear RNA processing, altering cellular energy states and activating pathways associated with inflammation and cancer. Models like the mouse prostate-specific *Elac2* knockout and the A537T substitution demonstrate how *ELAC2* disruptions facilitate cancer progression by modifying cellular metabolism and stress responses, particularly through altered processing of tRNAs and miRNAs. At the same time, however, reduction of *hoe-1* activity in *C. elegans*^124^ leads to cell cycle arrest rather than increased proliferation typical of cancer phenotypes. This discrepancy raises questions about whether the observed effects are due to RNAi-mediated knockdown rather than DNA mutations. Similar to *ELAC2*, mutations in *PRORP*, observed in both mouse^61^^,^^125^ (*Prorp* EX3DEL) and fly^126^ (*mldr, causing* Y121D and W465R substitutions) models, lead to an accumulation of unprocessed mt-tRNA molecules and mitochondrial dysfunctions. They translate to developmental delays and cardiomyopathy.^61^^,^^126^ Altogether, the insights from these models provide a comprehensive framework for understanding the molecular basis of mitochondrial diseases in humans.

In the context of hematopoietic disorders, mutations in the *PARN* gene in humans, as has already been pointed out, lead to DKCB6 and PFBMFT4. This is mirrored by the essential role of *Parn* in embryonic viability observed in mice, highlighting its crucial function in maintaining telomere integrity.^127^^,^^128^ PFBMFT4 in humans is associated with a decreased production of blood cells, manifesting as anemia (a deficiency of red blood cells) and leukopenia (a deficiency of white blood cells), symptoms that are also observed in zebrafish models with targeted *parn* disruption.^129^ In these animal models, specifically, mice with *PARN* knockdown and zebrafish, decreased RNA stability and impaired hematopoietic RNA processing are observed, respectively. These defects hinder effective blood cell maturation, linking *PARN* dysfunction directly to the clinical features of patients.

The phenotypes observed in organisms like *C. elegans*,^130^ *D. melanogaster*,^131^ *M. musculus*,^80^^,^^132^^,^^133^ and *S. cerevisiae*,^134^ due to mutations or depletions in *RNASEH1*-related genes, reveal a pattern of developmental disruption, mitochondrial dysfunction, and lethality, mirroring aspects of CPEO in humans.^110^ In *Rnaseh1*-deficient mice, a lack of the gene causes abnormal vacuoles and cristae structure, stalled DNA replication, and cell death. This leads to reduced mitochondrial DNA, buildup of DNA fragments, inefficient energy production systems, and reduced ATP generation. These similarities, especially in mitochondrial dysfunction and developmental delays, highlight a conserved role of RNase H1 in mitochondrial DNA maintenance and the regulation of gene expression across species.

## Concluding remarks

Model organisms, even those more evolutionarily distant, can be extremely useful in unraveling the background of human Mendelian diseases resulting from defects in RNases. Although certain aspects of these disorders are well characterized, many questions remain about the relevance of profound changes in the pool of RNA molecules for the molecular mechanisms and pleiotropic manifestations. Further research in this direction requires the use of organisms, not just cell lines or organoids, for a comprehensive understanding of the observed phenomena in genuine *in vivo* systems. Many monogenic mutations causing congenital diseases remain overlooked due to the lethality of the causal mutations. However, the scientific community's interest in model organism biology will undoubtedly facilitate the discovery of these genes, including RNases, associated with rare genetic disorders that exhibit lethal phenotypes in humans.

Beyond functional genetic screens, the integration of findings from various high-throughput omics platforms has emerged as a powerful tool in genetic disease-modeling endeavors. Such approaches have the potential to illuminate the epigenetic, genetic, transcriptomic, proteomic, and/or metabolomic aspects of a particular disease, offering a holistic perspective and aiding in the future modeling of the disease in a non-human model organism. Particularly important and promising are the rapidly developing single-cell approaches, which help to discern differential gene expression patterns specific to cell types and/or different stages of life. Such techniques can provide a clearer picture of the molecular landscape at a single-cell resolution, enabling the discovery of novel aspects of disease pathomechanisms and refining the previously established mechanistic overview. There is a surge in whole organism single-cell transcriptomic atlases for various non-human models like *C. elegans*, zebrafish, planarians, yeast, *Hydra*, frogs, and flies. These atlases help reveal spatiotemporal cellular heterogeneity and describe gene expression patterns. Possessing such detailed cellular, molecular, and genetic information about an organism can help assess its suitability as a model for particular disease scenarios.^135^ Furthermore, the utilization of high-throughput omics approaches will be instrumental in generating comprehensive datasets across organisms, enabling efficient disease mapping and prediction of vulnerable individuals within high-risk families, in the future.

## CRediT authorship contribution statement

**Annasha Dutta:** Writing – review & editing, Writing – original draft, Visualization, Investigation. **Anastasiia Zaremba:** Writing – review & editing, Writing – original draft, Visualization, Software, Investigation. **Paulina Jackowiak:** Writing – review & editing, Supervision, Project administration, Funding acquisition, Conceptualization.

## Data availability

Any additional information required to reanalyze the data reported in this paper is available from the lead contact upon request.

## Declaration of generative AI and AI-assisted technologies in the writing process

During the preparation of the original draft of this work, the authors used the free version of OpenAI-ChatGPT c2022 (v.GPT-4o, May 2024) to improve manuscript readability and language. After using this tool, the authors thoroughly reviewed and modified the manuscript, and the authors will take full responsibility for the content of the publication. The literature survey was done only by the authors, with no input from the AI tools.

## Funding

This work was financed by the National Science Centre, Poland (No. 2019/35/B/NZ2/02658 to P.J.).

## Conflict of interests

The authors have no competing interests to declare.

## Appendix ASupplementary data

Table S1 Selected RNase sequences, mutation effects, and disease correlation across species. The Excel sheet contains extensive information about RNase sequences from representative species, including their common names, protein sequences, IDs and names, gene symbols, associated diseases, reported alleles, homology status according to Alliance of Genome Resources, and the effects of RNase disruption. Table S1

## References

[1] Zuo Y., Deutscher M.P. (2001). Exoribonuclease superfamilies: structural analysis and phylogenetic distribution. Nucleic Acids Res.

[2] Li W.M., Barnes T., Lee C.H. (2010). Endoribonucleases: enzymes gaining spotlight in mRNA metabolism. FEBS J.

[3] Dixon H.B.F. (1998). Nomenclature Committee of IUBMB (NCIUBMB) and IUPAC-IUBMB joint commission on biochemical nomenclature (JCBN). Chem Int Newsmag IUPAC.

[4] Kim W.C., Lee C.H. (2009). The role of mammalian ribonucleases (RNases) in cancer. Biochim Biophys Acta.

[5] Amberger J.S., Bocchini C.A., Schiettecatte F., Scott A.F., Hamosh A. (2015). OMIM.org: online Mendelian Inheritance in Man (OMIM®), an online catalog of human genes and genetic disorders. Nucleic Acids Res.

[6] Bamshad M.J., Nickerson D.A., Chong J.X. (2019). Mendelian gene discovery: fast and furious with No end in sight. Am J Hum Genet.

[7] Baldridge D., Wangler M.F., Bowman A.N. (2021). Model organisms contribute to diagnosis and discovery in the undiagnosed diseases network: current state and a future vision. Orphanet J Rare Dis.

[8] Rohman M.S., Koga Y., Takano K., Chon H., Crouch R.J., Kanaya S. (2008). Effect of the disease-causing mutations identified in human ribonuclease (RNase) H_2_ on the activities and stabilities of yeast RNase H_2_ and archaeal RNase HII. FEBS J.

[9] Henras A.K., Sam M., Hiley S.L. (2005). Biochemical and genomic analysis of substrate recognition by the double-stranded RNA binding domain of yeast RNase III. RNA.

[10] Spudich G.M., Miller E.J., Marqusee S. (2004). Destabilization of the *Escherichia coli* RNase H kinetic intermediate: switching between a two-state and three-state folding mechanism. J Mol Biol.

[11] Catala M., Lamontagne B., Larose S., Ghazal G., Elela S.A. (2004). Cell cycle-dependent nuclear localization of yeast RNase III is required for efficient cell division. Mol Biol Cell.

[12] Nagirnaja L., Mørup N., Nielsen J.E. (2021). Variant*PNLDC1*, defective PiRNA processing, and azoospermia. N Engl J Med.

[13] Chial H. (2008). Mendelian genetics: patterns of inheritance and single-gene disorders. Nature Education.

[14] Wu Y., Zhang J., Peng B. (2019). Generating viable mice with heritable embryonically lethal mutations using the CRISPR-Cas9 system in two-cell embryos. Nat Commun.

[15] Jeong H.S., Backlund P.S., Chen H.C., Karavanov A.A., Crouch R.J. (2004). RNase H_2_ of *Saccharomyces cerevisiae* is a complex of three proteins. Nucleic Acids Res.

[16] Williams J.S., Kunkel T.A. (2014). Ribonucleotides in DNA: origins, repair and consequences. DNA Repair.

[17] Amon J.D., Koshland D. (2016). RNase H enables efficient repair of R-loop induced DNA damage. eLife.

[18] Kundnani D.L., Yang T., Gombolay A.L. (2024). Distinct features of ribonucleotides within genomic DNA in Aicardi-Goutières syndrome ortholog mutants of *Saccharomyces cerevisiae*. iScience.

[19] Goutières F., Aicardi J., Barth P.G., Lebon P. (1998). Aicardi-Goutières syndrome: an update and results of interferon-alpha studies. Ann Neurol.

[20] Aicardi J., Goutières F. (1984). A progressive familial encephalopathy in infancy with calcifications of the basal ganglia and chronic cerebrospinal fluid lymphocytosis. Ann Neurol.

[21] Hiller B., Achleitner M., Glage S., Naumann R., Behrendt R., Roers A. (2012). Mammalian RNase H_2_ removes ribonucleotides from DNA to maintain genome integrity. J Exp Med.

[22] Lyons S.M., Fay M.M., Akiyama Y., Anderson P.J., Ivanov P. (2017). RNA biology of angiogenin: current state and perspectives. RNA Biol.

[23] Li S., Hu G.F. (2010). Angiogenin-mediated rRNA transcription in cancer and neurodegeneration. Int J Biochem Mol Biol.

[24] Emara M.M., Ivanov P., Hickman T. (2010). Angiogenin-induced tRNA-derived stress-induced RNAs promote stress-induced stress granule assembly. J Biol Chem.

[25] Li S., Hu G.F. (2012). Emerging role of angiogenin in stress response and cell survival under adverse conditions. J Cell Physiol.

[26] Aluri K.C., Salisbury J.P., Prehn J.H.M., Agar J.N. (2020). Loss of angiogenin function is related to earlier ALS onset and a paradoxical increase in ALS duration. Sci Rep.

[27] Thiyagarajan N., Ferguson R., Subramanian V., Acharya K.R. (2012). Structural and molecular insights into the mechanism of action of human angiogenin-ALS variants in neurons. Nat Commun.

[28] van Es M.A., Diekstra F.P., Veldink J.H. (2009). A case of ALS-FTD in a large FALS pedigree with a K17I ANG mutation [published correction appears in *Neurology*. 2009;72(8):774]. Neurology.

[29] Hogg M.C., Rayner M., Susdalzew S. (2020). 5'ValCAC tRNA fragment generated as part of a protective angiogenin response provides prognostic value in amyotrophic lateral sclerosis. Brain Commun.

[30] Ye Z., Jin H., Qian Q. (2015). Argonaute 2: a novel rising star in cancer research. J Cancer.

[31] Cheloufi S., Dos Santos C.O., Chong M.M., Hannon G.J. (2010). A dicer-independent miRNA biogenesis pathway that requires Ago catalysis. Nature.

[32] Lessel D., Zeitler D.M., Reijnders M.R.F. (2020). Germline AGO2 mutations impair RNA interference and human neurological development. Nat Commun.

[33] Wu D., Yu W., Kishikawa H. (2007). Angiogenin loss-of-function mutations in amyotrophic lateral sclerosis. Ann Neurol.

[34] Rabe B. (2013). Aicardi-Goutières syndrome: clues from the RNase H_2_ knock-out mouse. J Mol Med (Berl)..

[35] Wu L., Xu Y., Zhao H., Li Y. (2020). RNase T2 in inflammation and cancer: immunological and biological views. Front Immunol.

[36] Caputa G., Zhao S., Criado A.E., Ory D.S., Duncan J.G., Schaffer J.E. (2016). RNASET2 is required for ROS propagation during oxidative stress-mediated cell death. Cell Death Differ.

[37] Haud N., Kara F., Diekmann S. (2011). rnaset2 mutant zebrafish model familial cystic leukoencephalopathy and reveal a role for RNase T2 in degrading ribosomal RNA. Proc Natl Acad Sci U S A.

[38] Liu Y., Zou W., Yang P. (2018). Autophagy-dependent ribosomal RNA degradation is essential for maintaining nucleotide homeostasis during *C. elegans* development. eLife.

[39] Benitez-Guijarro M., Lopez-Ruiz C., Tarnauskaitė Ž. (2018). RNase H_2_, mutated in Aicardi-Goutières syndrome, promotes LINE-1 retrotransposition. EMBO J.

[40] Majoor-Krakauer D., Willems P.J., Hofman A. (2003). Genetic epidemiology of amyotrophic lateral sclerosis. Clin Genet.

[41] Seven Y.B., Mitchell G.S. (2019). Mechanisms of compensatory plasticity for respiratory motor neuron death. Respir Physiol Neurobiol.

[42] Crow Y.J., Manel N. (2015). Aicardi–Goutières syndrome and the type I interferonopathies. Nat Rev Immunol.

[43] Cristini A., Tellier M., Constantinescu F. (2022). RNase H_2_, mutated in Aicardi-Goutières syndrome, resolves co-transcriptional R-loops to prevent DNA breaks and inflammation. Nat Commun.

[44] Tardieu M. (2010). Paediatric neurology: brain development at an interface between genetics, the environment, and the immune system. Lancet Neurol.

[45] Graves M.C., Fiala M., Dinglasan L.A. (2004). Inflammation in amyotrophic lateral sclerosis spinal cord and brain is mediated by activated macrophages, mast cells and T cells. Amyotroph Lateral Scler.

[46] Gamarra M., de la Cruz A., Blanco-Urrejola M., Baleriola J. (2021). Local translation in nervous system pathologies. Front Integr Neurosci.

[47] Bradshaw W.J., Rehman S., Pham T.T. (2017). Structural insights into human angiogenin variants implicated in Parkinson's disease and Amyotrophic Lateral Sclerosis. Sci Rep.

[48] Huang J., Liu P., Wang G. (2018). Regulation of mitochondrion-associated cytosolic ribosomes by mammalian mitochondrial ribonuclease T2 (RNASET2). J Biol Chem.

[49] Aryal N.K., Pant V., Wasylishen A.R. (2019). Dicer1 phosphomimetic promotes tumor progression and dissemination. Cancer Res.

[50] Meiklejohn K.M., Darbinyan A., Barbieri A.L. (2022). A review of DICER1: structure, function and contribution to disease. Diagn Histopathol.

[51] Choo K.B., Soon Y.L., Nguyen P.N., Hiew M.S., Huang C.J. (2014). MicroRNA-5p and -3p co-expression and cross-targeting in colon cancer cells. J Biomed Sci.

[52] Klein S.D., Martinez-Agosto J.A. (2020). Hotspot mutations in DICER1 causing GLOW syndrome-associated macrocephaly via modulation of specific microRNA populations result in the activation of PI3K/ATK/mTOR signaling. MicroRNA.

[53] Masarweh K., Mordechai O., Gur M., Bar-Yoseph R., Bentur L., Ilivitzki A. (2023). Challenges in DICER1-associated lung disease. J Clin Med.

[54] da Costa P.J., Menezes J., Saramago M. (2019). A role for DIS3L2 over natural nonsense-mediated mRNA decay targets in human cells. Biochem Biophys Res Commun.

[55] Astuti D., Morris M.R., Cooper W.N. (2012). Germline mutations in DIS3L2 cause the Perlman syndrome of overgrowth and Wilms tumor susceptibility. Nat Genet.

[56] Pirouz M., Du P., Munafò M., Gregory R.I. (2016). Dis3l2-mediated decay is a quality control pathway for noncoding RNAs. Cell Rep.

[57] Täuber H., Hüttelmaier S., Köhn M. (2019). POLIII-derived non-coding RNAs acting as scaffolds and decoys. J Mol Cell Biol.

[58] Pirouz M., Wang C.H., Liu Q. (2020). The Perlman syndrome DIS3L2 exoribonuclease safeguards endoplasmic reticulum-targeted mRNA translation and calcium ion homeostasis. Nat Commun.

[59] Haack T.B., Kopajtich R., Freisinger P. (2013). ELAC2 mutations cause a mitochondrial RNA processing defect associated with hypertrophic cardiomyopathy. Am J Hum Genet.

[60] Kim Y.A., Kim Y.M., Lee Y.J., Cheon C.K. (2017). The First Korean case of combined oxidative phosphorylation deficiency-17 diagnosed by clinical and molecular investigation. Korean J Pediatr.

[61] Rackham O., Busch J.D., Matic S. (2016). Hierarchical RNA processing is required for mitochondrial ribosome assembly. Cell Rep.

[62] Hochberg I., Demain L.A.M., Richer J. (2021). Bi-allelic variants in the mitochondrial RNase P subunit PRORP cause mitochondrial tRNA processing defects and pleiotropic multisystem presentations. Am J Hum Genet.

[63] Siira S.J., Rossetti G., Richman T.R. (2018). Concerted regulation of mitochondrial and nuclear non-coding RNAs by a dual-targeted RNase Z. EMBO Rep.

[64] Ricarte-Filho J.C., Casado-Medrano V., Reichenberger E. (2023). *DICER1* RNase IIIb domain mutations trigger widespread miRNA dysregulation and MAPK activation in pediatric thyroid cancer. Front Endocrinol.

[65] Saoura M., Powell C.A., Kopajtich R. (2019). Mutations in ELAC2 associated with hypertrophic cardiomyopathy impair mitochondrial tRNA 3'-end processing. Hum Mutat.

[66] Klein S., Lee H., Ghahremani S. (2014). Expanding the phenotype of mutations in DICER1: mosaic missense mutations in the RNase IIIb domain of DICER1 cause GLOW syndrome. J Med Genet.

[67] Ryzhkova A.I., Sazonova M.A., Sinyov V.V. (2018). Mitochondrial diseases caused by mtDNA mutations: a mini-review. Ther Clin Risk Manag.

[68] Nanjappa D.P., Babu N., Khanna-Gupta A., O'Donohue M.F., Sips P., Chakraborty A. (2021). Poly(A)-specific ribonuclease (PARN): more than just "mRNA stock clearing". Life Sci..

[69] Son A., Park J.E., Kim V.N. (2018). PARN and TOE1 constitute a 3' end maturation Module for nuclear non-coding RNAs. Cell Rep.

[70] Shukla S., Parker R. (2017). PARN modulates Y RNA stability and its 3'-end formation. Mol Cell Biol.

[71] Moon D., Segal M., Boyraz B. (2015). Mutations in the poly(A)-specific ribonuclease (PARN) impair telomerase RNA 3' end maturation in dyskeratosis congenita patients. Blood.

[72] Tummala H., Walne A., Collopy L. (2015). Poly(A)-specific ribonuclease deficiency impacts telomere biology and causes dyskeratosis congenita. J Clin Investig.

[73] Courtwright A.M., El-Chemaly S. (2019). Telomeres in interstitial lung disease: the short and the long of it. Ann Am Thorac Soc.

[74] Mills E.W., Wangen J., Green R., Ingolia N.T. (2016). Dynamic regulation of a ribosome rescue pathway in erythroid cells and platelets. Cell Rep.

[75] Ver Donck F., Ramaekers K., Thys C. (2023). Ribosome dysfunction underlies SLFN14-related thrombocytopenia. Blood.

[76] Stapley R.J., Pisareva V.P., Pisarev A.V., Morgan N.V. (2020). *SLFN14* gene mutations associated with bleeding. Platelets.

[77] Fletcher S.J., Pisareva V.P., Khan A.O., Tcherepanov A., Morgan N.V., Pisarev A.V. (2018). Role of the novel endoribonuclease SLFN14 and its disease-causing mutations in ribosomal degradation. RNA.

[78] Suzuki Y., Holmes J.B., Cerritelli S.M. (2010). An upstream open reading frame and the context of the two AUG codons affect the abundance of mitochondrial and nuclear RNase H1. Mol Cell Biol.

[79] Reyes A., Melchionda L., Nasca A. (2015). RNASEH1 mutations impair mtDNA replication and cause adult-onset mitochondrial encephalomyopathy. Am J Hum Genet.

[80] Misic J., Milenkovic D., Al-Behadili A. (2022). Mammalian RNase H1 directs RNA primer formation for mtDNA replication initiation and is also necessary for mtDNA replication completion. Nucleic Acids Res.

[81] Chen T.H., Koh K.Y., Lin K.M., Chou C.K. (2022). Mitochondrial dysfunction as an underlying cause of skeletal muscle disorders. Int J Mol Sci.

[82] Ezelle H.J., Malathi K., Hassel B.A. (2016). The roles of RNase-L in antimicrobial immunity and the cytoskeleton-associated innate response. Int J Mol Sci.

[83] Yin H., Jiang Z., Wang S., Zhang P. (2019). IFN-γ restores the impaired function of RNase L and induces mitochondria-mediated apoptosis in lung cancer. Cell Death Dis.

[84] Le Roy F., Salehzada T., Bisbal C., Dougherty J.P., Peltz S.W. (2005). A newly discovered function for RNase L in regulating translation termination. Nat Struct Mol Biol.

[85] Banerjee S., Li G., Li Y. (2015). RNase L is a negative regulator of cell migration. Oncotarget.

[86] Xiang Y., Wang Z., Murakami J. (2003). Effects of RNase L mutations associated with prostate cancer on apoptosis induced by 2',5'-oligoadenylates. Cancer Res.

[87] Anastasakis D., Skeparnias I., Shaukat A.N. (2016). Mammalian PNLDC1 is a novel poly(A) specific exonuclease with discrete expression during early development. Nucleic Acids Res.

[88] Ding D., Liu J., Dong K. (2017). PNLDC1 is essential for piRNA 3' end trimming and transposon silencing during spermatogenesis in mice. Nat Commun.

[89] Wei C., Yan X., Mann J.M. (2023). PNLDC1 catalysis and postnatal germline function are required for piRNA trimming, LINE1 silencing, and spermatogenesis in mice. bioRxiv.

[90] Maurer K.J., Quimby F.W. (2015). Laboratory Animal Medicine.

[91] Bellen H.J., Tong C., Tsuda H. (2010). 100 years of *Drosophila* research and its impact on vertebrate neuroscience: a history lesson for the future. Nat Rev Neurosci.

[92] Capecchi M.R. (2005). Gene targeting in mice: functional analysis of the mammalian genome for the twenty-first century. Nat Rev Genet.

[93] Izumi N., Shoji K., Sakaguchi Y. (2016). Identification and functional analysis of the pre-piRNA 3' trimmer in silkworms. Cell.

[94] Phillips K.A., Bales K.L., Capitanio J.P. (2014). Why primate models matter. Am J Primatol.

[95] Bult C.J., Sternberg P.W. (2023). The alliance of genome resources: transforming comparative genomics. Mamm Genome.

[96] Kettwig M., Ternka K., Wendland K. (2021). Interferon-driven brain phenotype in a mouse model of RNaseT2 deficient leukoencephalopathy. Nat Commun.

[97] Goh E., Okamura K. (2019). Hidden sequence specificity in loading of single-stranded RNAs onto *Drosophila* Argonautes. Nucleic Acids Res.

[98] Cai Q., Sun Y., Huang X. (2008). The *Caenorhabditis elegans* PcG-like gene sop-2 regulates the temporal and sexual specificities of cell fates. Genetics.

[99] Finger F., Ottens F., Hoppe T. (2021). The argonaute proteins ALG-1 and ALG-2 are linked to stress resistance and proteostasis. MicroPubl Biol..

[100] Duan Y., Li L., Panzade G.P., Piton A., Zinovyeva A., Ambros V. (2024). Modeling neurodevelopmental disorder-associated human AGO1 mutations in Caenorhabditis elegans Argonaute alg-1. Proc Natl Acad Sci U S A.

[101] Sievers F., Wilm A., Dineen D. (2011). Fast, scalable generation of high-quality protein multiple sequence alignments using Clustal Omega. Mol Syst Biol.

[102] Bodenhofer U., Bonatesta E., Horejš-Kainrath C., Hochreiter S. (2015). msa: an R package for multiple sequence alignment. Bioinformatics.

[103] UniProt Consortium (2023). UniProt: the universal protein knowledgebase in 2023. Nucleic Acids Res.

[104] Yu G., Smith D.K., Zhu H., Guan Y., Lam T.T. (2017). Ggtree: an R package for visualization and annotation of phylogenetic trees with their covariates and other associated data. Methods Ecol Evol.

[105] Chang W., Cheng J., Allaire J. (2024). shiny: web application framework for R. R package version 1.10.0.9000.

[106] Pokatayev V., Hasin N., Chon H. (2016). RNase H_2_ catalytic core Aicardi-Goutières syndrome-related mutant invokes cGAS-STING innate immune-sensing pathway in mice. J Exp Med.

[107] Potenski C.J., Epshtein A., Bianco C., Klein H.L. (2019). Genome instability consequences of RNase H_2_ Aicardi-Goutières syndrome alleles. DNA Repair.

[108] Chon H., Sparks J.L., Rychlik M. (2013). RNase H_2_ roles in genome integrity revealed by unlinking its activities. Nucleic Acids Res.

[109] Reijns M.A., Jackson A.P. (2014). Ribonuclease H_2_ in health and disease. Biochem Soc Trans.

[110] Hamosh A., Scott A.F., Amberger J., Valle D., McKusick V.A. (2000). Online Mendelian inheritance in man (OMIM). Hum Mutat.

[111] Weber T., Schlotawa L., Dosch R. (2020). Zebrafish disease model of human RNASET2-deficient cystic leukoencephalopathy displays abnormalities in early microglia. Biol Open.

[112] Hamilton N., Rutherford H.A., Petts J.J. (2020). The failure of microglia to digest developmental apoptotic cells contributes to the pathology of RNASET2-deficient leukoencephalopathy. Glia.

[113] Thompson D.M., Parker R. (2009). The RNase Rny1p cleaves tRNAs and promotes cell death during oxidative stress in *Saccharomyces cerevisiae*. J Cell Biol.

[114] Lee Y.S., Nakahara K., Pham J.W. (2004). Distinct roles for *Drosophila* dicer-1 and dicer-2 in the siRNA/miRNA silencing pathways. Cell.

[115] Banerjee A., Roy J.K. (2017). Dicer-1 regulates proliferative potential of *Drosophila* larval neural stem cells through bantam miRNA based down-regulation of the G1/S inhibitor Dacapo. Dev Biol.

[116] Wienholds E., Koudijs M.J., van Eeden F.J., Cuppen E., Plasterk R.H. (2003). The microRNA-producing enzyme Dicer1 is essential for zebrafish development. Nat Genet.

[117] Chen J.F., Murchison E.P., Tang R. (2008). Targeted deletion of Dicer in the heart leads to dilated cardiomyopathy and heart failure. Proc Natl Acad Sci USA.

[118] Hébert S.S., Papadopoulou A.S., Smith P. (2010). Genetic ablation of Dicer in adult forebrain neurons results in abnormal tau hyperphosphorylation and neurodegeneration. Hum Mol Genet.

[119] Towler B.P., Pashler A.L., Haime H.J. (2020). Dis3L2 regulates cell proliferation and tissue growth through a conserved mechanism. PLoS Genet.

[120] Hunter R.W., Liu Y., Manjunath H. (2018). Loss of *Dis3l2* partially phenocopies Perlman syndrome in mice and results in up-regulation of *Igf2* in nephron progenitor cells. Genes Dev.

[121] Wu D., Pedroza M., Chang J., Dean J. (2023). DIS3L2 ribonuclease degrades terminal-uridylated RNA to ensure oocyte maturation and female fertility. Nucleic Acids Res.

[122] Migunova E., Rajamani S., Bonanni S., Wang F., Zhou C., Dubrovsky E.B. (2023). Cardiac RNase Z edited via CRISPR-Cas9 drives heart hypertrophy in *Drosophila*. PLoS One.

[123] Stentenbach M., Ermer J.A., Rudler D.L. (2023). Multi-omic profiling reveals an RNA processing rheostat that predisposes to prostate cancer. EMBO Mol Med.

[124] Smith M.M., Levitan D.J. (2004). The *Caenorhabditis elegans* homolog of the putative prostate cancer susceptibility gene *ELAC2* hoe-1, plays a role in germline proliferation. Dev Biol.

[125] Kuznetsova I., Siira S.J., Shearwood A.J., Ermer J.A., Filipovska A., Rackham O. (2017). Simultaneous processing and degradation of mitochondrial RNAs revealed by circularized RNA sequencing. Nucleic Acids Res.

[126] Saoji M., Petersen C.E., Sen A., Tripoli B.A., Smyth J.T., Cox R.T. (2022). Reduction of *Drosophila* mitochondrial RNase P in skeletal and heart muscle causes muscle degeneration, cardiomyopathy, and heart arrhythmia. Front Cell Dev Biol.

[127] Benyelles M., Episkopou H., O'Donohue M.F. (2019). Impaired telomere integrity and rRNA biogenesis in PARN-deficient patients and knock-out models. EMBO Mol Med.

[128] Lee J.E., Lee J.Y., Trembly J., Wilusz J., Tian B., Wilusz C.J. (2012). The PARN deadenylase targets a discrete set of mRNAs for decay and regulates cell motility in mouse myoblasts. PLoS Genet.

[129] Dhanraj S., Gunja S.M., Deveau A.P. (2015). Bone marrow failure and developmental delay caused by mutations in poly(A)-specific ribonuclease (PARN). J Med Genet.

[130] Howe K., Davis P., Paulini M. (2012). WormBase: Annotating many nematode genomes. Worm.

[131] Filippov V., Filippov M., Gill S.S. (2001). *Drosophila* RNase H1 is essential for development but not for proliferation. Mol Genet Genom.

[132] Cerritelli S.M., Frolova E.G., Feng C., Grinberg A., Love P.E., Crouch R.J. (2003). Failure to produce mitochondrial DNA results in embryonic lethality in Rnaseh1 null mice. Mol Cell.

[133] Holmes J.B., Akman G., Wood S.R. (2015). Primer retention owing to the absence of RNase H1 is catastrophic for mitochondrial DNA replication. Proc Natl Acad Sci U S A.

[134] Cherry J.M., Hong E.L., Amundsen C. (2012). *Saccharomyces* Genome Database: the genomics resource of budding yeast. Nucleic Acids Res.

[135] Pisco A.O., Tojo B., McGeever A. (2021). Single-cell analysis for whole-organism datasets. Annu Rev Biomed Data Sci.

